# Synthesis, Conformational Analysis and Evaluation of the 2-aryl-4-(4-bromo-2-hydroxyphenyl)benzo[1,5]thiazepines as Potential α-Glucosidase and/or α-Amylase Inhibitors

**DOI:** 10.3390/molecules27206935

**Published:** 2022-10-16

**Authors:** Jackson K. Nkoana, Marole M. Maluleka, Malose J. Mphahlele, Richard M. Mampa, Yee Siew Choong

**Affiliations:** 1 Department of Chemistry, Faculty of Science and Agriculture, School of Physical and Mineral Sciences, University of Limpopo, Private Bag X1106, Sovenga 0727, South Africa; 2 Department of Chemistry, College of Science, Engineering and Technology, University of South Africa, Private Bag X06, Floridapark 1710, South Africa; 3 Institute for Research in Molecular Medicine (INFORMM), Universiti Sains Malaysia, Minden 11800, Penang, Malaysia

**Keywords:** 2,3-dihydrobenzo[*b*][1,5]thiazepines, X-ray structure, DFT, α-glucosidase, α-amylase, computational studies

## Abstract

The ambident electrophilic character of the 5-bromo-2-hydroxychalcones and the binucleophilic nature of 2-aminothiophenol were exploited to construct the 2-aryl-4-(4-bromo-2-hydroxyphenyl)benzo[1,5]thiazepines. The structures and conformation of these 2-aryl-4-(4-bromo-2-hydroxyphenyl)benzo[1,5]thiazepines were established with the use of spectroscopic techniques complemented with a single crystal X-ray diffraction method. Both ^1^H-NMR and IR spectroscopic techniques confirmed participation of the hydroxyl group in the intramolecular hydrogen-bonding interaction with a nitrogen atom. SC-XRD confirmed the presence of a six-membered intramolecularly hydrogen-bonded pseudo-aromatic ring, which was corroborated by the DFT method on **2b** as a representative example in the gas phase. Compounds **2a** (Ar = -C_6_H_5_), **2c** (Ar = -C_6_H_4_(4-Cl)) and **2f** (Ar = -C_6_H_4_(4-CH(CH_3_)_2_) exhibited increased inhibitory activity against α-glucosidase compared to acarbose (IC_50_ = 7.56 ± 0.42 µM), with IC_50_ values of 6.70 ± 0.15 µM, 2.69 ± 0.27 µM and 6.54 ± 0.11 µM, respectively. Compound **2f**, which exhibited increased activity against α-glucosidase, also exhibited a significant inhibitory effect against α-amylase (IC_50_ = 9.71 ± 0.50 µM). The results of some computational approaches on aspects such as noncovalent interactions, calculated binding energies for α-glucosidase and α-amylase, ADME (absorption, distribution, metabolism and excretion) and bioavailability properties, gastrointestinal absorption and blood–brain barrier permeability are also presented.

## 1. Introduction

**Figure 1:**
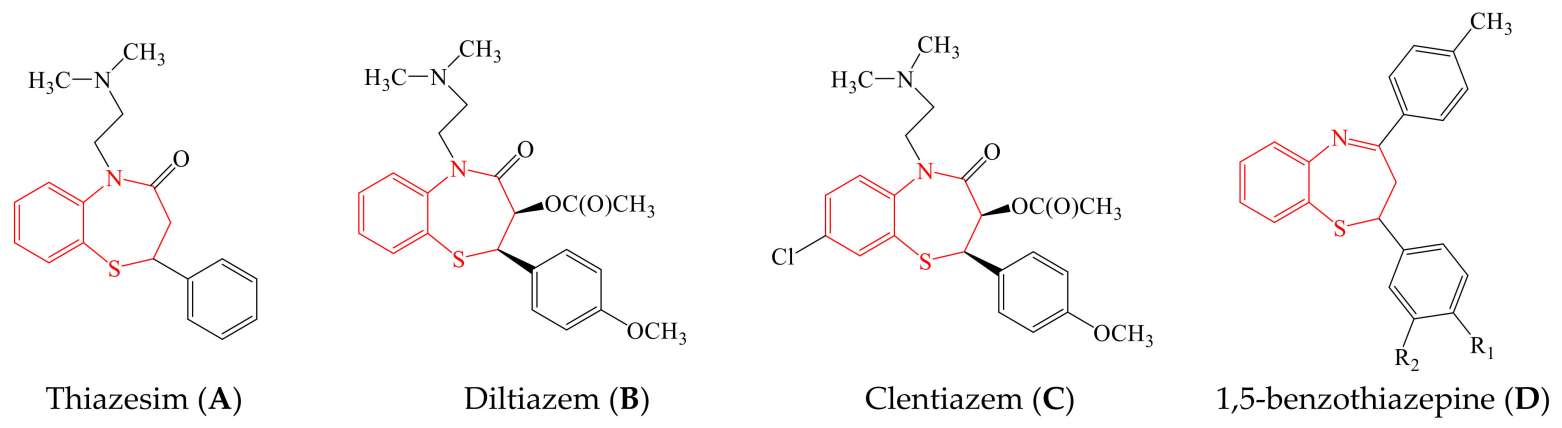
Structures of examples of the medicinally important 1,5-benzothiazepine derivatives.

The 1,5-benzothiazepine nucleus is present in a number of clinically used drugs with a broad spectrum of biological and pharmacological activities, and examples include diltiazem, clentiazem, thiazesim, quetiapine, and clothiapine [1]. Their therapeutic properties can be traced back to the entry in the market of the antidepressant drug thiazesim (**A**), shown in Figure 1 [2]. Two of the 1,5-benzothiazepine derivatives, namely, diltiazem (**B**) and clentiazem (**C**), are known for their cardiovascular action [1,2]. Though the cardiovascular and central nervous system are the two major areas for the clinical use of benzodiazepine analogues, other biological targets for the benzothiazepines have been explored to establish their anti-inflammatory, antihypertensive, antibacterial, antifungal, anticancer, diuretic and antidepressant properties [1,3,4]. The 2,3-dihydro-1,5-benzothiazepines, in particular, have also received attention as new classes of cholinesterase, adenosine kinase, MAP kinase protein and glycogen synthase kinase-3β (GSK-3β) inhibitors [1]. GSK-3β plays an important role in diabetes and neurodegenerative diseases such as dementia and Alzheimer’s disease [5]. A recent structure–activity relationship (SAR) study on a series of the 2,4-diaryl-2,3-dihydro-1,5-benzothiazepines of the generalised structure **D**, shown in Figure 1, revealed that the benzothiazepine ring plays a significant role in α-glucosidase inhibition [6]. α-Glucosidase is a membrane-bound carbohydrate-hydrolysing enzyme found in the epithelial wall of the small intestine. Inhibitors of this enzyme’s activity play a crucial role in the treatment of type 2 diabetes mellitus (T2DM) by decreasing the glucose level in the bloodstream [6]. Salivary α-amylase starts to breakdown the α-(1,4)-glycosidic bonds of starch into monosaccharides during digestion, and this slow process continues in the upper part of the small bowel where the pH allows optimal activity of the pancreatic α-amylase to convert large starch and glycogen molecules into simpler absorbable sugars. These sugars are, in turn, converted into glucose by the intestinal α-glucosidase and transported across the intestinal mucosa to blood vessels for distribution into cells and tissues [7]. This process results in an increased level of glucose in the blood, also known as postprandial hyperglycaemia (PPHG), which is a primary indication for T2DM. Dual inhibition of the activity of α-glucosidase and α-amylase would suppress carbohydrate digestion, delay glucose uptake and result in reduced blood sugar levels [8]. Controlled hyperglycaemia prevents the complications of T2DM such as stroke, cardiovascular heart diseases and nonvascular pathologies such as cancer [9].

Several researchers continue to study the conformations and crystalline structures of benzothiazepine analogues to explore noncovalent (intramolecular and intermolecular) interactions, control molecular conformations and improve their physicochemical properties (durability, solubility and bioavailability) as drug molecules [10,11,12]. As part of our renewed interest in the conformation [13] and biological activity of benzoheterazepines [14], we decided to synthesize a series of 2,4-diaryl-substituted 2,3-dihydrobenzo[*b*][1,5]thiazepines based on the 5-bromo-2-hydroxychalcones as the scaffold for a one-pot acid-catalysed thia-Michael addition and cyclization with 2-aminothiophenol. This rational design was inspired by the literature precedents that established the electron-withdrawing inductive effect of the bromine atom could help the drug molecules in forming hydrogen- and/or halogen-bonding interactions with the receptors that stabilize the interactions between drug molecules and the protein targets and enhance biological activity [15,16,17]. Moreover, the proximity of the 4-bromo-2-hydroxyphenyl ring to the nitrogen atom on the target 2,3-dihydrobenzo[*b*][1,5]thiazepine scaffold is envisaged to facilitate intramolecular hydrogen bonding with the hydroxyl group. The intramolecular hydrogen-bonding interaction has been found to facilitate alignment of the drug molecule in the protein pocket, resulting in increased ligand–receptor interactions [18]. It was envisioned that the presence of the lipophilic bromine atom and potential hydrogen-bonding hydroxyl group as well as the presence of various substituents on the 2-phenyl ring on the benzo[1,5]thiazepine framework could increase the inhibitory effect of these compounds against α-glucosidase and/or α-amylase activities. First, we studied the conformation of these benzodiazepine analogues in solution and a solid state by means of spectroscopic and single-crystal X-ray diffraction (XRD) techniques complemented with the density functional theory (DFT) method in the gas phase. Intermolecular interactions in the crystal structure of a representative example of the test compounds were studied in detail using computational techniques. Moreover, their biological activity as potential antidiabetic agents has been evaluated through enzymatic assays in vitro against α-glucosidase and α-amylase activities. These experimental results were complemented with a molecular docking (in silico) study in the binding pockets of these carbohydrate-hydrolysing enzymes. The key aspects of the pharmacokinetics of the most active compounds from each series, namely, the absorption, distribution, metabolism and excretion (ADME) properties, have also been evaluated to predict their drug likeness. Moreover, a molecular dynamics study was conducted on a representative compound with high affinity for α-glucosidase and α-amylase binding sites in an attempt to correlate the results of in silico and in vitro studies.

## 2. Results and Discussion

### 2.1. Chemical Synthesis and Spectroscopic Analyses

**Scheme 1:**
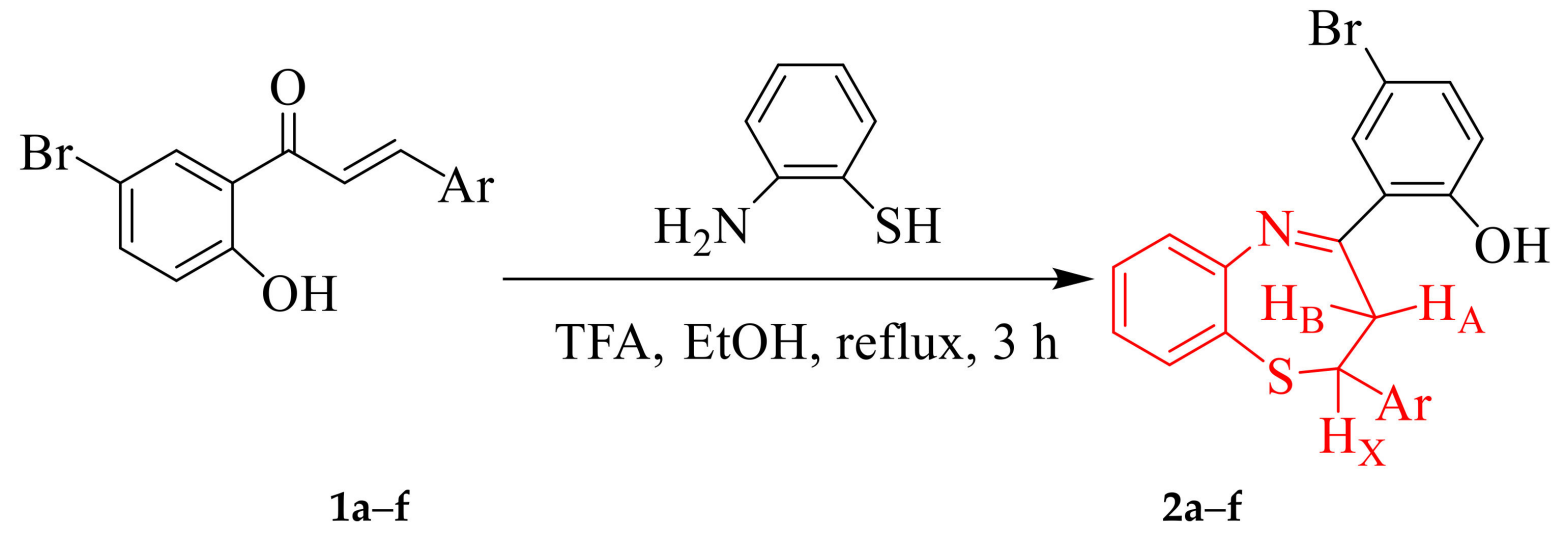
Heteroannulation of **1a**–**f** with 2-aminothiophenol to provide **2a**–**f**.

The test compounds were prepared as shown in Scheme 1 and the designation of substituents and percentage yields of the compounds are listed in Table 1. The 5-bromo-2-hydroxychalcones **1a**–**f** used as substrates in this investigation were prepared, in turn, via base-mediated Claisen–Schmidt aldol condensation of 5-bromo-2-hydroxyacetophenone (1 equiv.) and the corresponding benzaldehyde derivative (1 equiv.). Their melting point values and spectroscopic data were found to compare favourably with the literature data [19,20]. Although there are several methods available to construct 1,5-benzothiazepine skeletons [21,22], a very typical method involves the reaction of 2-aminothiophenol derivatives with α,β-unsaturated esters, α,β-unsaturated ketones or chalcones under neutral, acidic and basic conditions [6,12,22,23,24,25]. The synthesis of 1,5-benzothiazepines **2a**–**f** involved condensing the 5-bromo-2-hydroxychalcones **1a**–**f** with 2-aminothiophenol in the presence of trifluoroacetic acid (TFA) in ethanol under reflux for 3 h. Mechanistically, the reaction proceeds through the initial thia-Michael addition of the thiol group to the enone functionality of the chalcone, which is followed by the cyclization of the incipient intermediate to provide the corresponding 1,5-benzothiazepine in a single-pot operation [24]. The structure of the achieved compounds was clarified by a combination of ^1^H-NMR, ^13^C-NMR, IR and mass spectral data (Figure S1 in Supplementary Materials).

The ^1^H-NMR spectral splitting patterns and the vicinal coupling constant values of these compounds were used to investigate the conformational preferences of the dihydrobenzothiazepine ring in CDCl_3_ solution. The aliphatic region of the ^1^H-NMR spectra of these compounds at 400 MHz represents an ABX system with the two anisochronous protons of the methylene (CH_2_) group and the methine (CH) proton of the 7-membered heterocyclic ring, each resonating as a doublet of doublets (dd) in the regions δ 3.52–3.05–3.52 ppm and δ 4.99–5.02 ppm, respectively. The corresponding protons in the case of the ^1^H-NMR spectra of the analogous 4-(2,4-dibromo-1,3-dihydroxyphenyl)-substituted 2,3-dihydrobenzo[*b*][1,5]thiazepines acquired in CDCl_3_ at 500 MHz were previously reported to resonate as a doublet around δ = 3.32 ppm for the CH_2_ group and a triplet around δ = 4.98 ppm for the methine proton [25]. The methylene protons are chemically nonequivalent in any conformation of the dihydrobenzothiazepine system and couple with each other (*J*_gem_), and each couples differently to the vicinal methine proton (*J*_vic_ ≠ *J*_gem_). The set of multiplets corresponding to the aliphatic protons of the test compounds were assigned with the aid of 2D nuclear Overhauser effect spectroscopy (NOESY) and heteronuclear multiple-bond correlation experiment (HMBC) spectroscopy on **2d** as a representative model (refer to Figure S2 in SI). A significant downfield shift of the resonance corresponding to H_B_ to around δ 3.52 ppm is ascribed to the magnetic anisotropy of the parallel 1,2 and 4,5 bonds in analogy with the literature precedent for the 1,5-benzoheterazepine analogues [26]. The signal for the OH group resonates significantly downfield around δ = 14.60 ppm as a less intense and slightly broad singlet, which is consistent with the participation of this hydrogen atom in the intramolecular hydrogen-bond interaction with a nitrogen atom to form a six-membered chair-like pseudo-ring. The O–H stretching bands in the infrared spectra of the test compounds recorded using the thin-film method and represented in Figure S1 of SI, on the other hand, are generally inconspicuous, which is consistent with the definition of a strong intramolecular bond [27]. We were able to obtain single crystals of a representative compound **2b** and the geometry of these compounds was distinctly confirmed using the XRD method.

### 2.2. Solid-State Structural Analysis Using X-ray Crystallography

**Figure 2:**
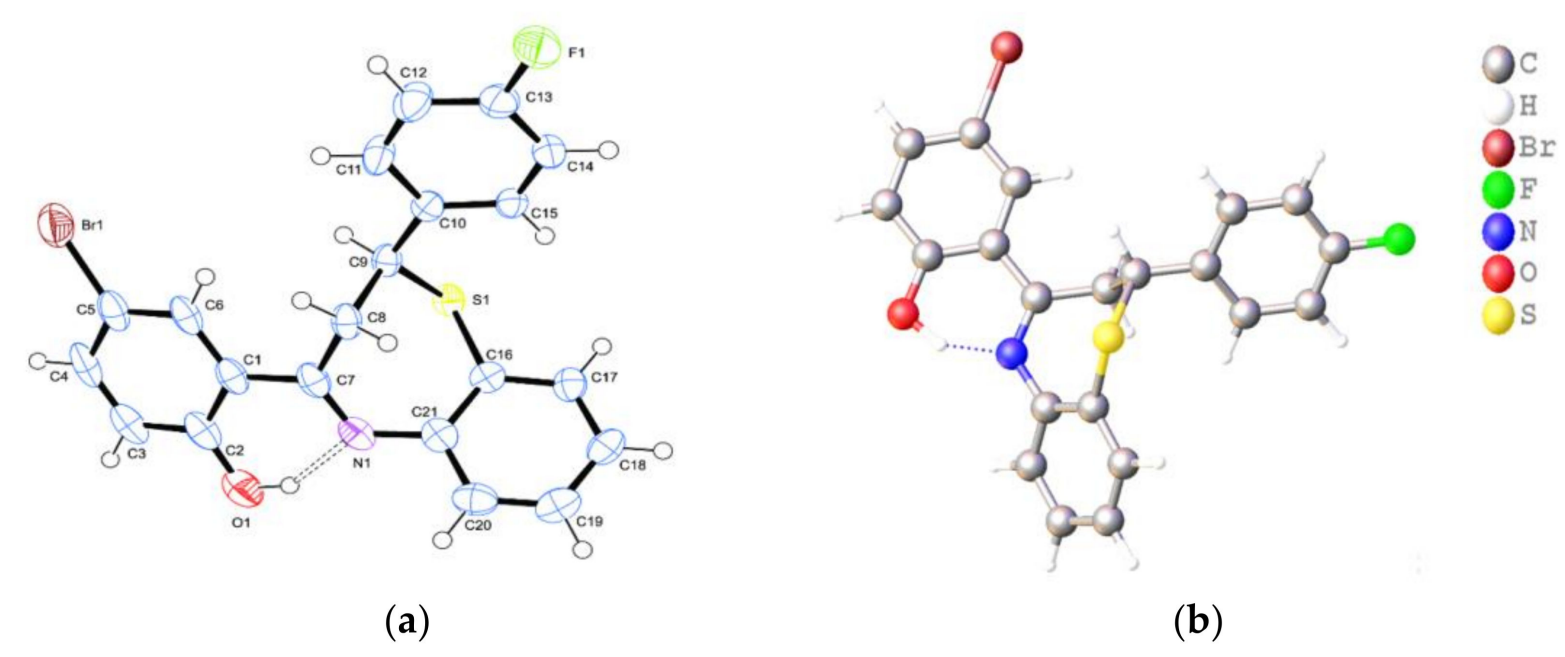
ORTEP diagram of **2b** with thermal ellipsoids drawn at 50% probability level (**a**) and its optimized geometry (**b**) at the B3LYP/LANL2DZ level. The atom-labelling scheme for this compound is based on the XRD structure and the numbering differs from the systematic one.

XRD analysis of **2b** confirmed the existence of one molecule in the asymmetric unit (Figure 2) with the intramolecular hydrogen bond involving the hydroxyl group (donor) and nitrogen atom (acceptor) with a hydrogen bond distance, d(N(1)⋯H(1)⋯O(1) = 2.551(5) Å, to form a six-membered quasi-aromatic chelate ring. The seven-membered ring deviates from the coplanarity of the fused benzo ring and the dihedral angles of the three protons of the AMX system are 179.0° and 62.58°, which represent an almost perfect staggered conformation. The gas-phase computation at the B3LYP/LANL2DZ level of theory also confirmed the presence of a six-membered intramolecular hydrogen-bonded pseudo-ring (Figure 2b), thus corroborating the solution phase and the solid-state experiments. The RMSD (root mean-square deviation) between the X-ray and optimized structure (in the gas phase) of **2b** is 1.360 Å, and this value indicates that the two structures superimposed very well.

**Figure 3:**
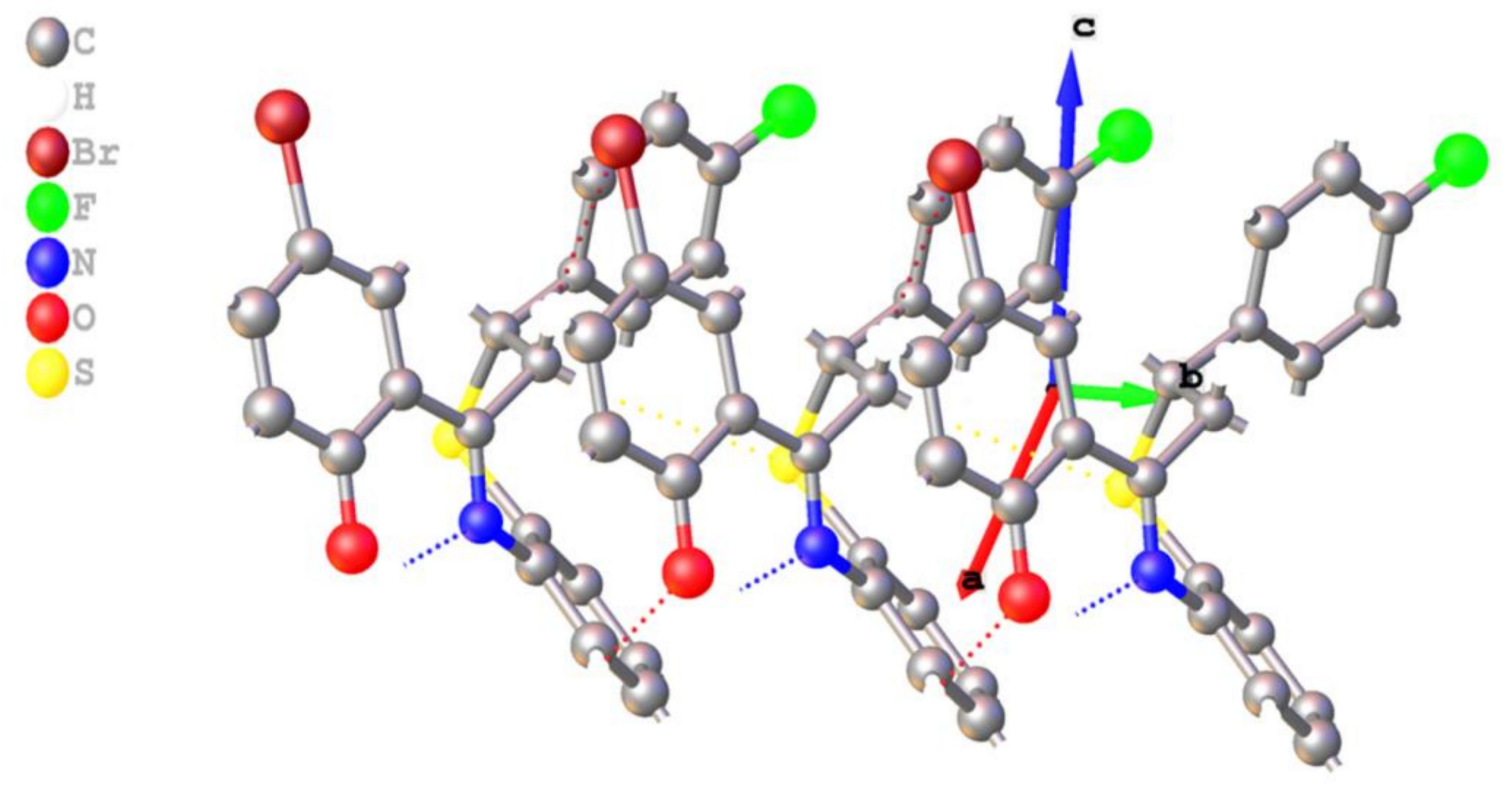

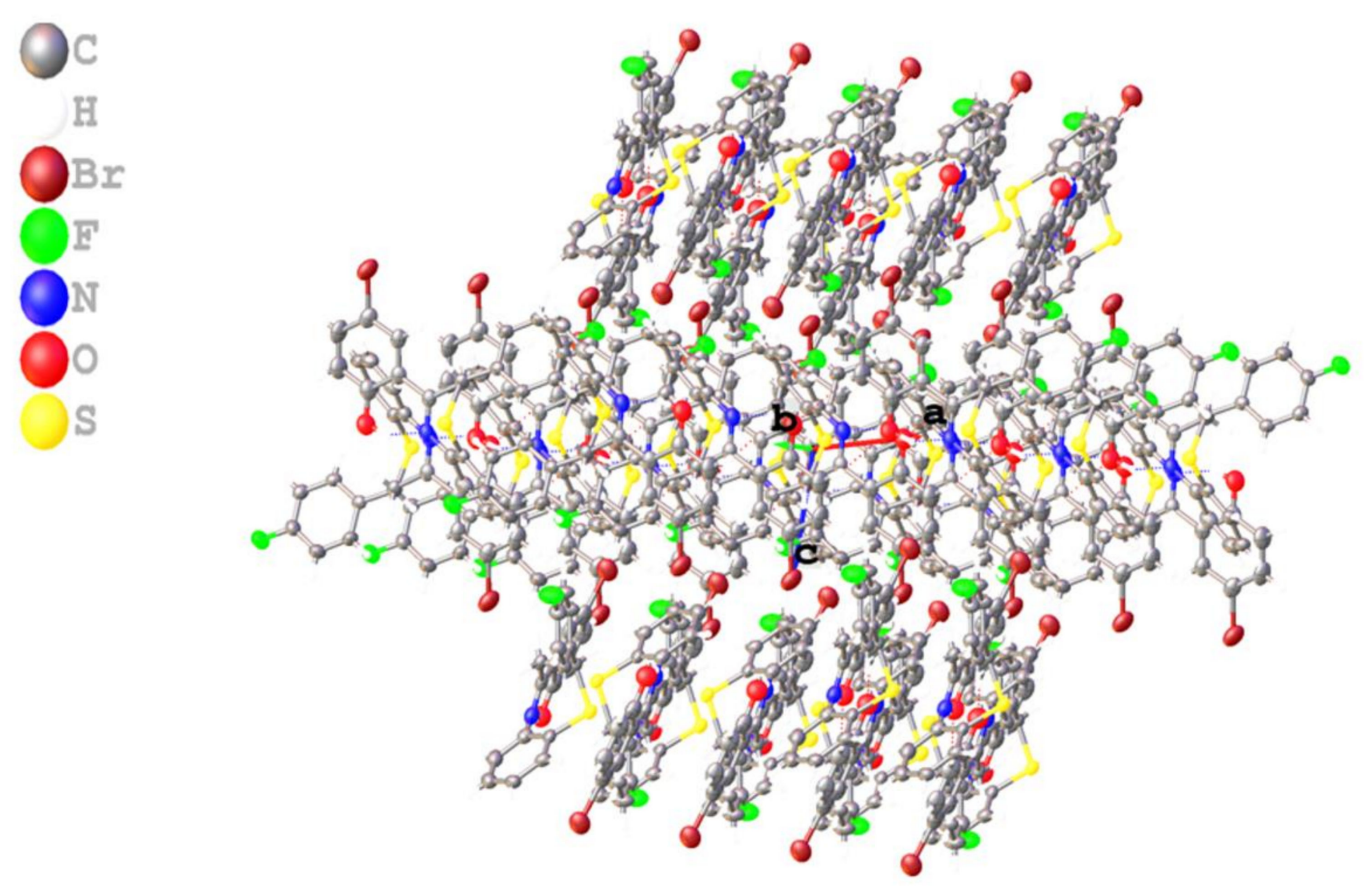
Perspective view of the crystal structure of **2b** showing the intermolecular interactions along the *b*-axis.

Hydrogen bonds (inter- and intramolecular), halogen bonds and aromatic–aromatic (π···π, C–H···π stacking) interactions, as well as other weak contacts such as the van der Waals or dipole–dipole, play important roles in material science and medicinal chemistry [16,28]. These noncovalent interactions are of interest to the medicinal chemists to tailor the physical and chemical stability, solubility and bioavailability of drugs [29], and to extrapolate SAR [30]. The intramolecularly hydrogen-bonded molecules of **2a** are held together in a chain along the b-axis by weak intermolecular hydrogen bonds of the type C–H⋯O contacts (Figure 3). The oxygen atom (O1) of the methoxy group and the *ortho* hydrogen atom (H3) of one molecule, for example, are involved in the weak intermolecular C–H⋯O hydrogen-bonding interaction with the corresponding atoms of the neighbouring molecule to form an eight-membered ring motif with an R^2^_2_(8) graph set. The neighbouring molecules are also linked together by short halogen–halogen-bonding interactions of the type F⋯F contacts to form a one-dimensional supramolecular structure along the crystallographic b-axis.

### 2.3. Computational Methods

**Figure 4:**
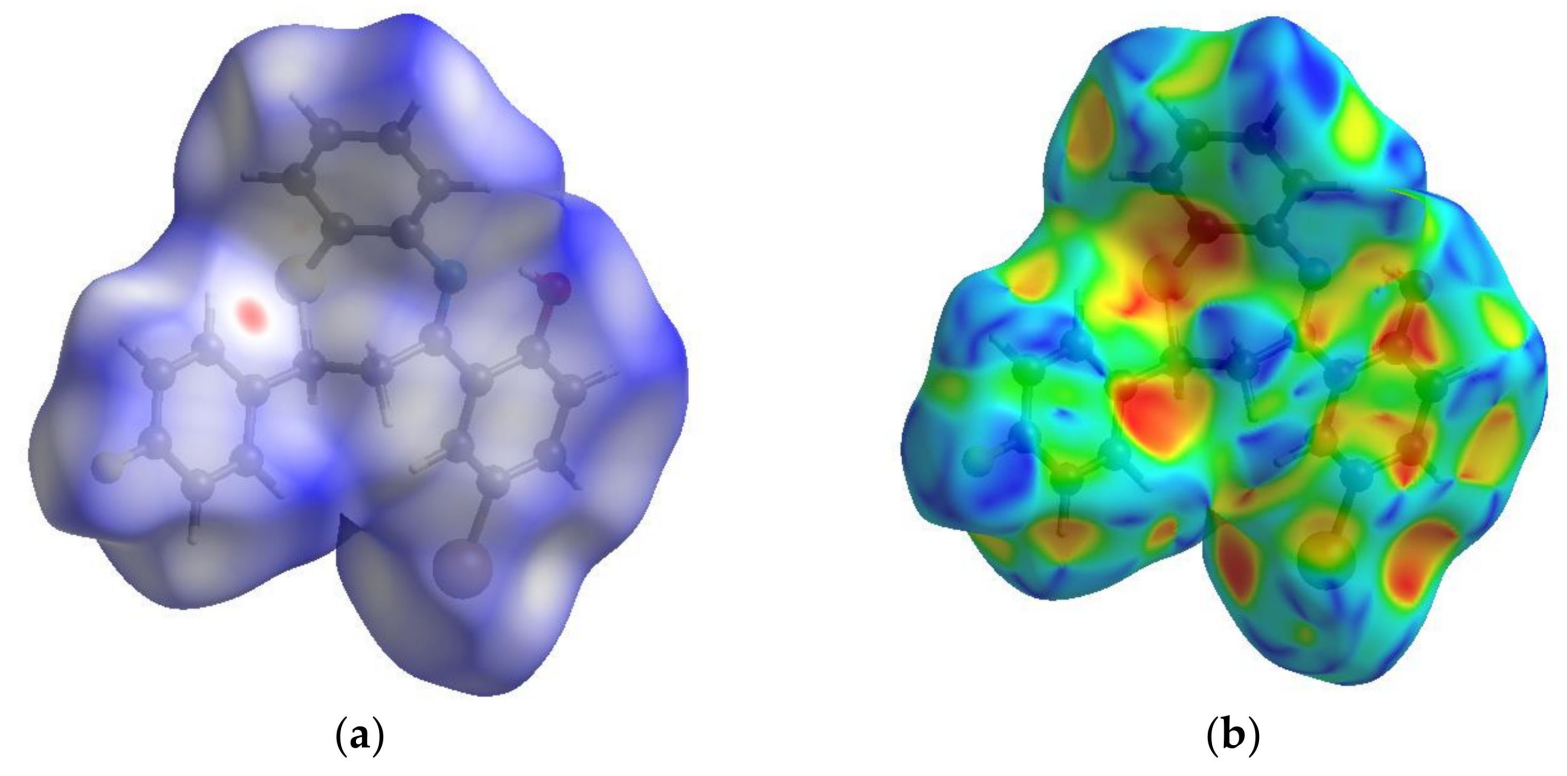
The *d*_norm_ surface (**a**) and shape-index surface (**b**) for **2b**.

**Figure 5:**
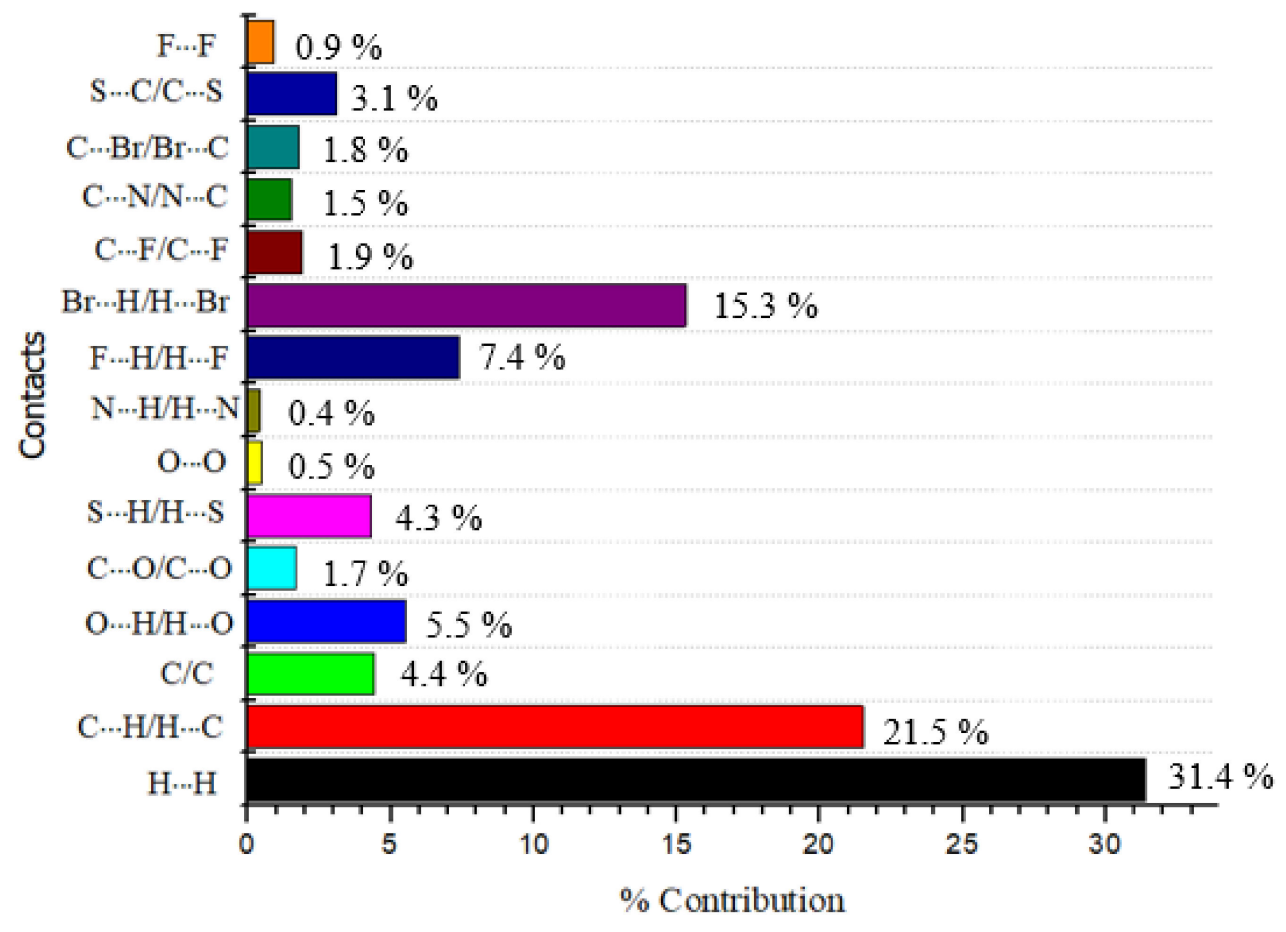
Quantification of the intermolecular interactions of **2b**.

The Hirshfeld surface (Figure 4a) and shape-index surface plots (Figure 4b) of compound **2b** were carried out using *CrystalExplorer* 17.5 software [31] based on the corresponding Common Internet File’s (CIF’s) input to obtain information about intermolecular contacts and their quantitative contribution to the supramolecular self-assembly. The plots suggest that the intermolecular H···H contacts make up 31.4% of the Hirschfeld surface, and these are the highest contributing contacts in the crystal packing of this compound (see Figure 5). The reciprocal C⋯H contacts, which contribute 21.5% toward the Hirshfeld surface, are attributed to C−H···π interactions. The C···C contacts represent the hydrophobic π⋯π interactions and these produce a minor contribution (4.4%) to the compound’s crystal packing. The presence of consecutive triangular regions of red and blue around the aromatic rings on the shape-index surface indicates a π⋯π stacking interaction. The other reciprocal intermolecular contacts, such as Br⋯H/H⋯Br, F⋯H/H⋯F, O⋯H/H⋯O and S⋯H/H⋯S contacts, contribute 15.3, 7.4, 5.5 and 4.3% to the total Hirschfeld surface, respectively, which also reveals the importance of these contacts in the molecular packing of the studied compounds. The H···O/O···H contacts, due to nonclassical intermolecular C–H···O hydrogen bonds, constitute 5.5% of the Hirshfeld surface of this compound. The anisotropic electronic charge distribution around covalently bound fluorine has previously been found to give rise to a positive σ-hole in its outer portion and a weaker positive or negative belt around its lateral sides [32]. These make this atom work as an XB donor in the gas, solution and solid phases. Both of the fluorine atoms in F_2_ have been found to possess positive σ-holes, which are also present in some organofluoro compounds in which fluorine is bound to O, N and/or C atoms [33]. The present study revealed the existence of weak halogen bonds of the types C⋯F (1.9%) and C⋯Br (1.8%), in which the halogen atoms act as XB donors and π electrons as acceptors. The noncovalent interactions, such as halogen–halogen and halogen-bonding interactions, are of interest in medicinal chemistry, chemical biology, supramolecular chemistry and crystal engineering [33]. At least in our opinion, the various noncovalent interactions observed for the test compounds should be valuable for rational drug design and help in understanding interactions of these halogenated ligands with proteins.

**Figure 6:**
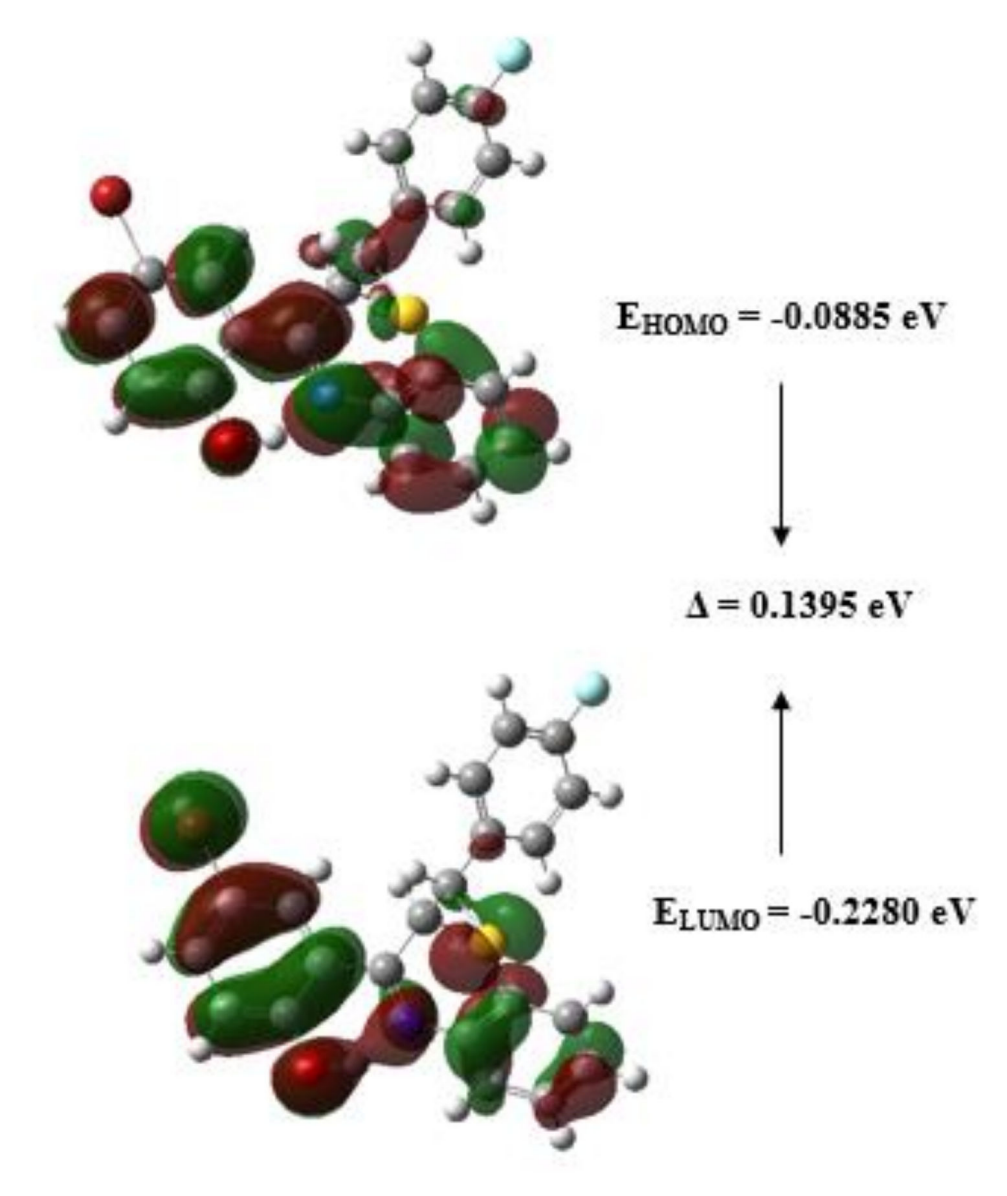
The HOMO–LUMO orbitals of **2b** using B3LYP/LANL2DZ level of theory.

A graphical representation of the distribution and energy levels for the highest occupied molecular orbital (HOMO) and the lowest unoccupied molecular orbital (LUMO) computed at the B3LYP/LANL2DZ level of theory for **2b** are shown in Figure 6 below. The HOMO is envisaged to be delocalized over the entire 3-phenyl-2,3-dihydrobenzothiazepine framework and partially on C-1, C-2 and C-4 of the 2-(4-fluorophenyl) substituent. The LUMO, on the other hand, is made up predominantly of the 3-phenyl-2,3-dihydrobenzothiazepine scaffold and a partial contribution from C-1 of the 2-(4-fluorophenyl) group. The frontier molecular orbitals are mainly composed of π-atomic orbitals, presumably due to the n-π∗ electronic transition. The conjugated molecule is characterised by a small HOMO–LUMO separation of 0.1395 eV, which would allow a significant intramolecular charge transfer from the electron-donor group (HOMO) to the electron-acceptor group (LUMO) through the π-conjugated path. The global reactivity parameters, such as the ionization potential (I = −E_HOMO_), electron affinity (A = −E_LUMO_), electronegativity (χ = −μ), chemical potential (μ = −(I + A)/2), global hardness (η = (I − A)/2) and softness (S = 1/η), and electrophilicity index (ω = μ2/2η) have also been calculated at the B3LYP/LANL2DZ level, and the corresponding data are summarized in Table 2. Chemical hardness (0.1395 eV) and chemical softness (7.692 eV) indicate that the molecule is kinetically stable. The low values of the chemical hardness (0.06747 eV) and HOMO–LUMO energy gap (0.1395 eV) indicate that this compound is reactive. The chemical potential value of 0.1583 eV and electrophilicity index value of 0.089 also support its reactivity as a good electrophile.

**Figure 7:**
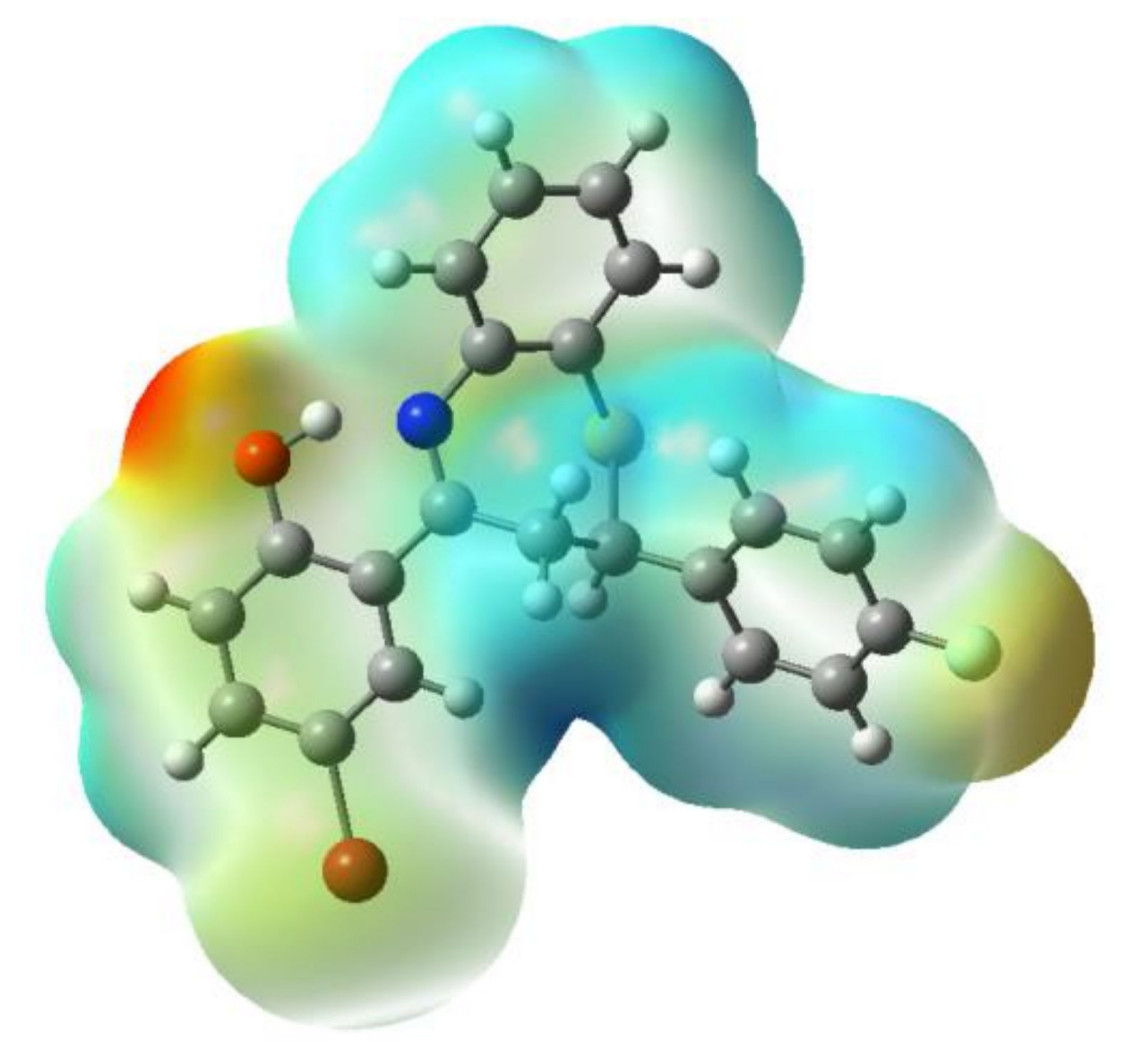
MEP map over the optimized structure of **2b** in increasing order: red < orange < yellow < green < blue.

The molecular electrostatic potential (MEP) of the optimized geometry of **2b** calculated at B3LYP/LANL2DZ, shown in Figure 7 below, provides a visual interpretation of the electronic density distribution within a molecule. MEP is useful in identifying sites for nucleophilic and electrophilic attack, as well as in studies of biological recognition and hydrogen-bonding interactions [34]. The positive (blue) potential sites are distributed around the hydrogen atoms and sulphur atom, and these are related to nucleophilic reactivity. The negative region (red), which is susceptible to electrophilic attack, is mainly located over the oxygen atom of the hydroxyl group and was caused by the contribution of the lone-pair electrons. These sites also give information about the region from where the compound can make intermolecular interactions.

Motivated by the recent biological activity study of the 2,3-dihydro-1,5-benzothiazepines as α-glucosidase inhibitors [6] and in continuation of our interest in antidiabetic agents, we decided to evaluate compounds **2a**–**f** through an enzymatic assay in vitro to study the inhibitory effect against α-glucosidase and α-amylase activities.

### 2.4. Biology

#### 2.4.1. Inhibitory Effect of **2a**–**f** on α-Glucosidase and α-Amylase

In vitro enzymatic assays were performed on the test compounds to evaluate their inhibitory effect against α-glucosidase and α-amylase using acarbose as the positive control. The inhibition curves for α-glucosidase and α-amylase with the test compounds are included in Figures S3 and S4 in the Supplementary Materials, and the results are reported as the concentration inhibiting 50% of the enzyme activity (IC_50_). The IC_50_ values (μM) represented in Table 3 were calculated from the log dose-inhibition curves and are expressed as means ± standard deviations (SD) of three independent experiments. The preliminary SAR of compounds **2a**–**f** has been rationalized with respect to the nature of the 2-aryl substituent (Ar). Compounds **2a** (Ar = -C_6_H_5_), **2c** (Ar = -C_6_H_4_(4-Cl)) and **2f** (Ar = -C_6_H_4_(4-CH(CH_3_)_2_) exhibited increased inhibitory activity against α-glucosidase compared to acarbose (IC_50_ = 7.56 ± 0.42 µM) and their IC_50_ values are 6.70 ± 0.15 µM, 2.69 ± 0.27 µM and 6.54 ± 0.11 µM, respectively. These compounds, with a significant inhibitory effect against α-glucosidase, could serve as the first-line drugs for the treatment of T2DM to prevent the digestion of carbohydrates in the intestine, defer glucose absorption and, in turn, suppress PPHG. Moreover, α-glucosidase inhibitors have promising therapeutic potential in the treatment of other disorders such as cancer, HIV, hepatitis, obesity, certain forms of dyslipidaemia and cardiovascular diseases [35]. However, benzothiazepine derivatives **2a** and **2c** were found to be moderately inhibiting against α-amylase compared to acarbose (IC_50_ = 2.63 ± 0.22 µM) with IC_50_ values of 20.87 ± 0.08 µM and 22.86 ± 0.04 µM, respectively. Compound **2f** substituted with an isopropyl group at the *para* position of the 2-aryl ring, which exhibited increased activity against α-glucosidase, also exhibited a significant inhibitory effect against α-amylase with an IC_50_ value of 9.71 ± 0.50 µM. The presence of a moderately π-electron-delocalizing 4-fluorophenyl group at the 2-position of the heterocyclic scaffold of **2b** resulted in a moderate inhibitory effect against α-glucosidase and α-amylase with IC_50_ values of 17.20 ± 0.07 µM and 15.85 ± 0.05 µM, respectively. The presence of a strong π-electron-delocalizing methoxy group on the *meta* or *para* position of the 2-phenyl ring of **2d** or **2e** also resulted in moderate activity against α-glucosidase (IC_50_ = 13.25 ± 1.00 µM or 15.87 ± 0.13 µM, respectively) and significantly reduced activity against α-amylase (IC_50_ = 46.16 ± 0.31 µM or 55.14 ± 0.19 µM, respectively) among the test compounds. Inhibitors of α-glucosidase with mild inhibitory activity against α-amylase, on the other hand, constitute one of the major therapeutic strategies for the treatment of T2DM. Compounds **2a** (selectivity index (SI) = IC_50_(α-glucosidase)/IC_50_(α-amylase) value of 0.11), **2c** (SI = 0.12) and **2f** (SI = 0.67) with an increased inhibitory effect against α-glucosidase and moderate activity against α-amylase will probably suppress carbohydrate digestion, delay glucose uptake and result in reduced blood sugar levels with minimal gastrointestinal side effects.

The binding interactions of the test compounds within the active site of α-glucosidase (PDB code: 5NN8) and α-amylase (PDB code: 5E0F) were simulated with the help of molecular docking studies.

#### 2.4.2. Molecular Docking Studies

The binding free energy calculations showed that all the test compounds have better binding affinity against both α-glucosidase and α-amylase compared to acarbose as the control (Table 4). Compounds **2c** and **2f** have a higher affinity toward α-glucosidase, which is consistent with their inhibitory effect against this enzyme. Similarly, compounds **2c** and **2f** are also among the highest binding ligands with α-amylase compared to acarbose. However, the docking simulation is a quantitative prediction, and hence, the obtained binding free energy sometimes might not correlate perfectly with the in vitro results. Nevertheless, the conformation and interaction of a compound in the binding site of the target protein could still be useful in the elucidation of SAR and for future ligand optimization.

**Figure 8:**
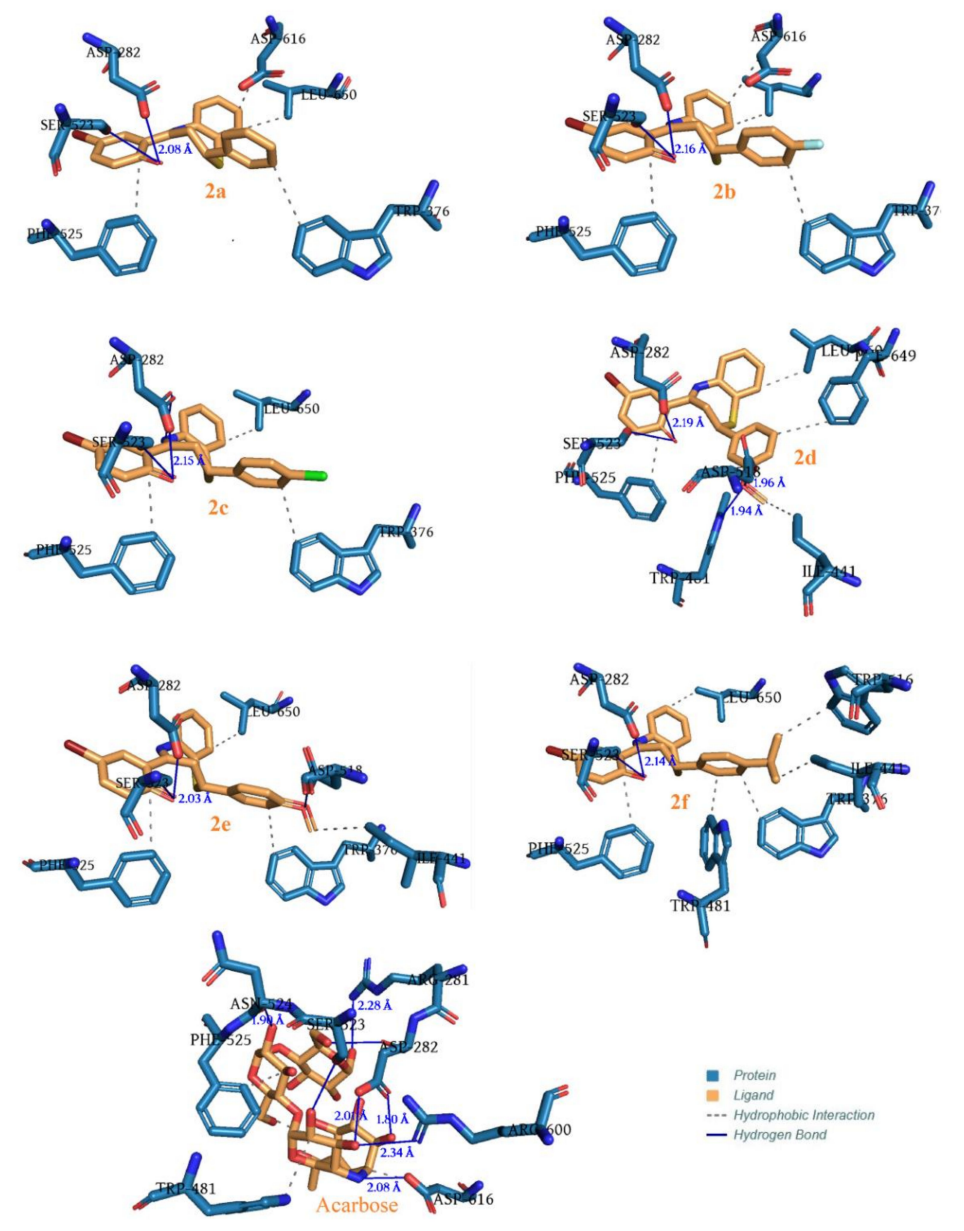
Interactions of **2a**–**f** and acarbose with protein residues in the binding pocket of α-glucosidase. The distances of strong hydrogen bonds (distance < 2.5 Å) are labelled in this figure.

Molecular docking (in silico) was conducted on these benzothiazepine derivatives against both enzymes to predict plausible protein–ligand interactions at a molecular level. The active site of α-glucosidase is made up of the protein residues, Trp376, Asp404, Trp516, Asp518, Met519, Arg600, Asp616, Phe649 and His674 [36]. The analysis showed that the binding of compounds **2a**–**f** with α-glucosidase is driven by hydrophobic interactions between the ligand and the protein residues Phe525 and Leu650, and hydrogen-bonding interactions with the residues Asp280 and Ser523 (Figure 8). Compound **2f** is predicted to form the highest number (6) of hydrophobic interactions with α-glucosidase, which is consistent with its binding affinity and inhibitory effect against this enzyme. Although acarbose forms a relatively higher number of hydrogen bonds with α-glucosidase, its binding affinity was less than that of the test compounds, probably due to its hydrophilic tetrasaccharide architecture compared to **2f** with a 4-(CH_3_)_2_CHC_6_H_4_- branch that provides hydrophobic contact with α-glucosidase.

**Figure 9:**
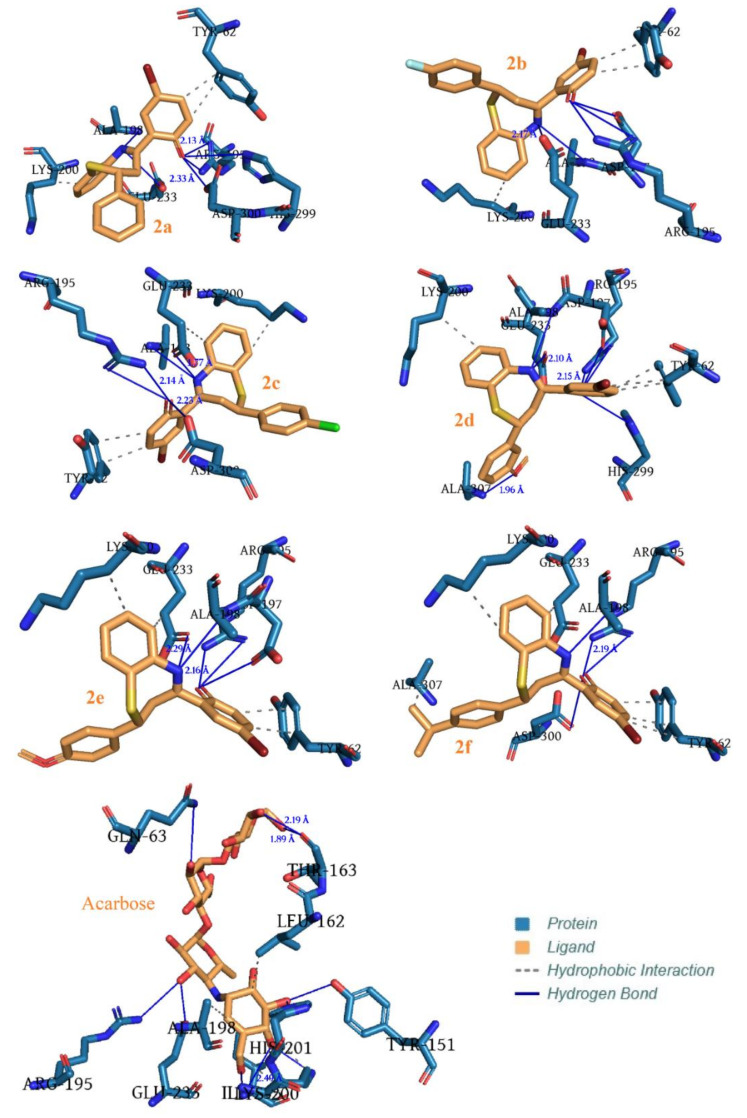
The interactions of compounds **2a**–**f** and acarbose in the binding pocket of α-amylase obtained from docking simulation. The distances of strong hydrogen bonds (distance < 2.5 Å) are labelled in this figure.

Compounds **2a**–**f**, which exhibited moderate activity against α-amylase, and comparable binding energies are predicted to be involved in 3–6 hydrophobic interactions with the protein residues Tyr62 and Lys200 (Figure 9). Hydrogen-bonding interactions are predicted between these compounds and the α-amylase residues Arg195 and Ala198, as well as Glu233 of the catalytic triad. The docked conformation of acarbose with α-amylase also yielded a relatively high number of hydrogen-bonding interactions. However, **2f** still exhibited significantly better affinity against α-amylase (slightly more than 2 kcal/mol of more negative binding energy) compared with acarbose. The architecture of compound **2f** might be the key functional group to provide a better binding with α-amylase as well as α-glucosidase due to the higher hydrophobic interactions of the isopropyl-benzene region with the proteins.

### 2.5. Drug-Likeness Predictions of ***2a***–***2f***

**Figure 10:**
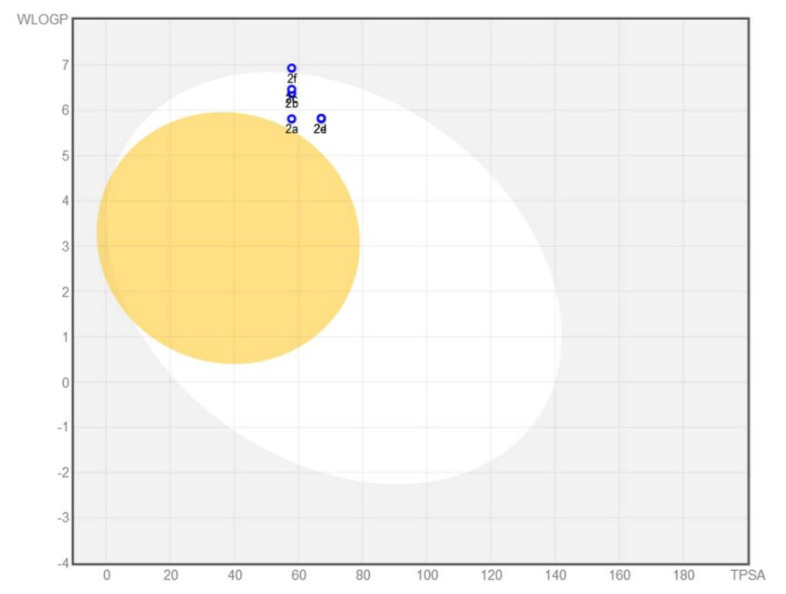
The Brain Or IntestinaL EstimateD permeation method (BOILED-Egg) for the prediction of the lipophilicity and polarity of compounds **2a**–**2f**.

The absorption, distribution, metabolism and excretion (ADME) properties of these compounds were established theoretically to predict their drug-likeness for future in vivo studies (Table 5). Pharmacokinetic property predictions showed that the partition coefficient (cLogP) of these test compounds is slightly greater than 5, which is in violation of Lipinski’s rule of five [37], suggesting poor permeability. This indicates that the test compounds are less water-soluble and might possess bioaccumulation activity. Blood–brain barrier (BBB) permeability and gastrointestinal absorption properties of the title compound were investigated by using the Brain Or IntestinaL EstimateD permeation method (BOILED-Egg) from SwissADME [38], and the diagram is represented in Figure 10. The molecules restricted to the yellow region can only cross the blood–brain barrier passively, and this is a major problem when designing central nervous system drugs [39]. Those compounds that fall only into the white region have a high chance to be absorbed by the gastrointestinal tract. The blue dot, on the other hand, shows the ability of a compound to be actively effluxed by the permeability glycoprotein (PGP). The latter plays an important role in drug transport to the organs and is involved in the determination of the bioavailability and drug’s pharmacokinetic properties such as absorption, distribution, elimination and drug–drug interactions [40]. Compounds that fall outside the white or yellow region indicate the unabsorbed and were not brain penetrant. The gastrointestinal absorption evaluation showed that compounds **2a**–**e** are highly possible in the passive absorption of the gastrointestinal tract and predicted as being actively effluxed by the permeability glycoprotein. Derivative **2f**, on the other hand, is predicted to exhibit slightly less passive absorption.

### 2.6. Functional Motions of α-Glucosidase and α-Amylase in Complex with Compound ***2f***

**Figure 11:**
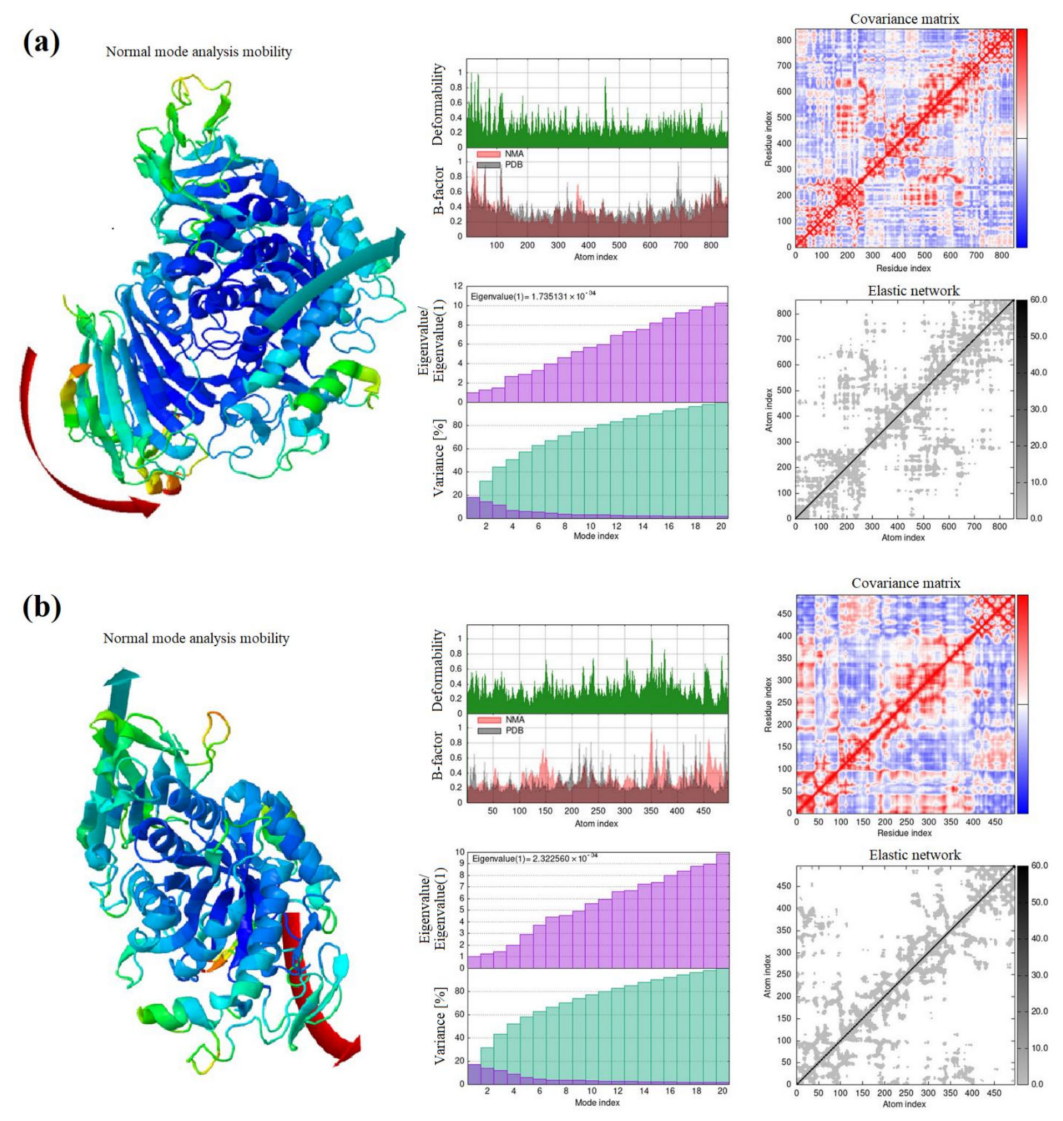
Normal mode analysis (NMA) of the mobility (arrows indicate the direction of the motion; coloured according to the NMA), deformability, B-factor, eigenvalues, variance associated with each normal mode, covariance matrix and elastic network of α-glucosidase (**a**) and α-glucosidase (**b**) in complex with compound **2f**.

The statistical structural flexibility simulation analysis on the docked complex of α-glucosidase and α-amylase with **2f** was performed using iMODS to predict the stability and flexibility of this compound when complexed with α-glucosidase and α-amylase. Normal mode analysis showed two main motilities of α-glucosidase and α-amylase upon binding with compound **2f** (Figure 11). In addition, both proteins also experience correlation (coloured red in the covariance matrix) and are rather flexible (coloured white in the elastic network map) when bound to compound **2f**. The low relative eigenvalues for α-glucosidase (1.74 × 10^−4^) and α-amylase (2.32 × 10^−4^) suggest that both systems are relatively stable. The stability of both proteins were further evidenced by main-chain deformability, B-factor and variance plots.

## 3. Experimental Procedure

### 3.1. Materials and Methods

Commercially available chemicals including solvents were obtained from Sigma-Aldrich (Modderfontein, Johannesburg, South Africa) and used without purification. The melting point values of the prepared compounds were recorded on a Stuart SMP10 melting point apparatus (Cole-Parmer, Stone, Staffordshire/UK). Their infrared (IR) spectra were recorded using the thin-film method on a Bruker VERTEX 70 FT-IR Spectrometer (Bruker Optics, Billerica, MA, USA) equipped with a diamond attenuated total reflectance (ATR) accessory. The ^1^H-NMR and ^13^C-NMR spectra of the prepared compounds were acquired at 300 K on a Bruker Avance 400 (400 MHz) spectrometer (Bruker Optics, Billerica, MA, USA), and the chemical shift values are reported in parts per million (ppm) relative to the central residual proton peak or to the central carbon peak of deuterated chloroform (CDCl_3_); 7.25 ppm and 77.0 ppm, respectively. Multiplicity was indicated as follows: s = singlet, d = doublet, t = triplet, sept = septet, m = multiplet, dd = doublet of doublet and td = triplet of doublet. High-resolution mass spectra (HR-MS) were recorded on an AB SCIEX X500 QTOF system (SCIEX, MA, USA) in positive and negative ESI mode. The ion-source temperature was set to 550 ℃ and IS voltage was set to 5500 V.

### 3.2. Typical Procedure for the Synthesis of the 5-Bromo-2-Hydroxychalcones (***1a***–***f***)

A stirred mixture of 5-bromo-2-hydroxyacetophenone (1 equiv.) and benzaldehyde derivative (1 equiv.) in ethanol at room temperature (RT) was treated dropwise with a 10% NaOH solution (2 mL/mmol of carbonyl derivative). The mixture was stirred at room temperature for 12 h and then quenched with ice-cold water. The precipitate was filtered, dissolved in chloroform and the organic solution was dried over anhyd. MgSO_4_. The salt was filtered off and the solvent was evaporated under reduced pressure. The analytical data for compounds **1a**–**f** were found to compare favourably with the literature data [19,20].

### 3.3. General Procedure for the Synthesis of Benzothiazepines ***2a***–***f***

A stirred mixture of chalcone **1** (3.00 mmol) and 2-aminothiophenol (3.6 mmol) in ethanol (40 mL) was treated dropwise with trifluoroacetic acid (0.5 equiv.). The mixture was stirred under reflux for 3 h with thin-layer chromatography (TLC) monitoring and then poured into ice-cold water. The precipitate was filtered on a sintered funnel and recrystallized from ethanol to provide **2**. The following compounds were prepared following this procedure.

4-Bromo-2-((*E*)-2,3-dihydro-2-phenylbenzo[*b*][1,5]thiazepine-4-yl)phenol (**2a**)



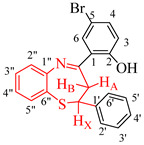



Yellow solid (2.31 g, 85%), mp. 128–137 ºC; IR (ATR): ν_max_ 478.9, 507.4, 938.9, 1120.3, 1328.7, 1578.7, 1871.65, 1899.3, 3015.3 cm^−1^; ^1^H-NMR (CDCl_3_, 400 MHz): δ 2.99 (dd, *J* = 12.4 and 13.2 Hz, 1H, H_A_), 3.23 (dd, *J* = 4.8 and 13.2 Hz, H_B_), 4.99 (dd, *J* = 4.8 and 12.1 Hz, 1H, H_X_), 6.88 (d, *J* = 8.8 Hz, 1H, H-3), 7.16 (td, *J* = 1.6 and 7.6 Hz, 1H, H-4″), 7.23 (m, 3H, Ar), 7.27 (m, 3H, Ar), 7.38 (dd, *J =* 2.4 and 8.8 Hz, 1H, H-5″), 7.42 (td, *J* = 1.4 and 7.8 Hz, 1H, H-3″), 7.51 (d, *J* = 2.4 Hz, 1H, H-6), 7.57 (dd, *J* = 1.2 and 7.7 Hz, 1H, H-2″), 14.50 (s, 1H, OH); ^13^C-NMR (CDCl_3_, 100 MHz): δ 36.8, 60.1, 110.1, 119.8, 120.6, 124.5, 125.7, 126.1, 126.8, 128.3, 129.0, 130.1, 130.8, 135.4, 136.2, 143.2, 148.4, 161.8, 172.3; HRMS (ES): MH^+^ calculated for C_21_H_17_BrNOS, 410.0133; found: 410.0125.

4-Bromo-2-((*E*)-2,3-dihydro-2-(4-fluorophenyl)benzo[*b*][1,5]thiazepine-4-yl)phenol (**2b**)



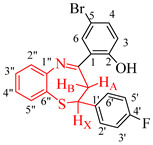



Yellow solid (1.23 g, 92%) mp. 128–134 ºC; IR (ATR): ν_max_ 461.5, 506.5, 624.9, 753.0, 826.3, 1155.0, 1219.1, 1296.1, 1338.1, 1476.0, 1551.7, 1597.0, 3051.2 cm^−1^; ^1^H-NMR (CDCl_3_, 400 MHz): δ 3.25 (dd, *J* = 11.6 and 13.4 Hz, 1H, H_A_), 3.52 (dd, *J* = 4.8 and 13.3 Hz, 1H, H_B_), 5.29 (dd, *J* = 4.8 and 11.9 Hz, 1H, H_x_), 7.19 (1H, d, *J* = 8.8 Hz, 1H, H-3), 7.25 (t, *J* = 8.6 Hz, 2H, H-3′,5′), 7.46 (td, *J* = 1.4 and 7.6 Hz, 1H, H-4″), 7.53 (m, 3H, H-4 and H-2′,6′), 7.68 (dd, *J* = 2.4 and 8.8 Hz, 1H, H-5″), 7.71 (td, *J =* 1.4 and 7.8 Hz, 1H, H-3″), 7.78 (d, *J* = 2.3 Hz, 1H, H-6), 7.86 (dd, *J* = 1.4 and 7.8 Hz, 1H, H-2″), 14.75 (s, 1H, OH); ^13^C-NMR (CDCl_3_, 100 MHz): δ 37.0, 59.3, 110.1, 115.8 (d, ^2^*J*_CF_ = 21.0 Hz), 119.7, 120.6, 124.2, 125.74, 126.7, 127.9 (d, ^3^*J*_CF_ = 8.0 Hz), 130.2, 130.8, 135.3, 136.3, 139.9 (d, ^4^*J*_CF_ = 3.0 Hz), 148.4, 162.8 (d, ^1^*J*_CF_ = 250.0 Hz), 172.0.; HRMS (ES): MH^+^ calculated for C_21_H_16_BrFNOS, 428.0173; found: 428.0145.

4-Bromo-2-((*E*)-2,3-dihydro-2-(4-chlorophenyl)benzo[*b*][1,5]thiazepine-4-yl)phenol (**2c**)



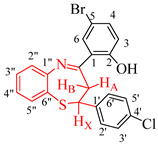



Yellow solid (1.27 g, 96%), mp. 167–172 °C); IR (ATR): ν_max_ 444.3, 499.2, 627.6, 756.9, 821.5, 864.3, 1088.3, 1206.5, 1253.0, 1335.8, 1474.4, 1551.5, 1597.1, 2989.4, 3070.0 cm^−1^; ^1^H-NMR (CDCl_3_, 400 MHz): δ 2.97 (dd, *J* = 12.1 and 13.2 Hz, 1H, H_A_), 3.25 (dd, *J* = 4.8 and 13.3 Hz, 1H, H_B_), 4.99 (dd, *J* = 4.8 and 12.0 Hz, 1H, H_X_), 6.92 (d, *J* = 8.8 Hz, 1H, H-3), 7.20 (m, 3H, H-2′,6′ and H-4″), 7.26 (m, 3H, H-4 and H-3′,5′), 7.42 (dd, *J* = 2.4 and 8.9 Hz, 1H, H-5″), 7.46 (td, *J* = 1.4 and 7.8 Hz, 1H, H-3″), 7.55 (d, *J* = 2.4 Hz, 1H, H-6), 7.58 (dd, *J* = 1.2 and 7.7 Hz, 1H, H-2″), 14.45 (s, 1H, OH); ^13^C-NMR (CDCl_3_, 100 MHz): δ 36.8, 59.1, 110.2, 119.7, 120.6, 124.0, 125.8, 126.9, 127.5, 129.1, 130.3, 130.7, 134.0, 135.3, 136.3, 141.6, 148.4, 161.8, 172.0; HRMS (ES): MH^+^ calculated for, C_21_H_16_BrClNOS, 443.0019; found: 443.0042.

4-Bromo-2-((*E*)-2,3-dihydro-2-(3-methoxyphenyl)benzo[*b*][1,5]thiazepine-4-yl)phenol (**2d**)



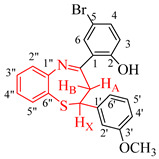



Yellow solid (1.17 g, 89%), mp. 161–167 °C; IR (ATR): ν_max_ 461.5, 559.2, 626.0, 697.6, 771.06, 819.8, 865.7, 1027.8, 1145.2, 1212.5, 1259.5, 1338.0, 1480.3, 1582.8, 2920.4, 3049 cm^−1^; ^1^H-NMR (CDCl_3_, 400 MHz): δ 3.06 (1H, dd, *J* = 12.0 and 13.3 Hz, H_A_), 3.31 (dd, *J* = 4.8 and 13.3 Hz, 1H, H_B_), 3.77 (s, 3H, -OCH_3_), 5.04 (dd, *J* = 4.8 and 12.0 Hz, 1H, H_x_), 6.84 (m, 2H, H-4′ and H-6′), 6.88 (m, 1H, H-2′), 6.96 (d, *J* = 8.8 Hz, 1H, H-3), 7.23 (td, *J* = 1.4 and 7.6 Hz, 1H, H-4″), 7.26 (m, 1H, H-5′), 7.30 (dd, *J* = 1.2 and 7.9 Hz, 1H, H-4), 7.47 (dd, *J* = 2.4 and 8.8 Hz, 1H, H-5″), 7.50 (td, *J* = 1.4 and 7.8 Hz, 1H, H-3″), 7.57 (d, *J* = 2.4 Hz, 1H, H-6), 7.65 (dd, *J* = 1.3 and 7.7 Hz, 1H, H-2″), 14.57 (s, 1H, OH); ^13^C-NMR (CDCl_3_, 100 MHz): δ 36.8, 55.3, 60.0, 110.1, 112.1, 113.4, 118.4, 119.8, 120.6, 124.5, 125.7, 126.8, 130.1, 130.1, 130.3, 135.3, 136.2, 144.6, 148.4, 159.9, 161.8, 172.3; HRMS (ES): MH^+^ calculated for, C_22_H_19_BrNO_2_S, 441.0028; found: 441.0221.

4-Bromo-2-((*E*)-2,3-dihydro-2-(4-methoxyphenyl)benzo[*b*][1,5]thiazepine-4-yl)phenol (**2e**)



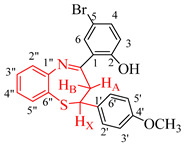



Yellow Solid (1.98 g, 99%), mp. 131–136 °C; IR (ATR): ν_max_ 461.1, 523.7, 625.5, 830.1, 1023.6, 1245.5, 1297.3, 1340.9, 1476.6, 1510.6, 1550.8, 1598.8, 2920.3, 3056.8 cm^−1^; ^1^H-NMR (CDCl_3_, 400 MHz): δ 3.05 (dd, *J* = 12.0 and 13.6 Hz, 1H, H_A_), 3.30 (dd, *J* = 4.8 and 13.2 Hz, 1H, H_B_), 3.80 (s, 3H, -OCH_3_), 5.08 (dd, *J* = 4.8 and 11.6 Hz, 1H, H_x_), 6.88 (m, 2H, H-3′,5′), 6.97 (d, *J* = 8.8 Hz, 1H, H-3), 7.24 (dd, *J =* 1.4 and 7.6, 1H, H-4″), 7.27 (m, *J* = 2.4 Hz, 2H, H-2′,6′), 7.32 (dd, *J* = 1.2 and 7.9 Hz, 1H, H-4), 7.46 (dd, *J* = 2.4 and 8.8 Hz, 1H, H-5″), 7.48 (td, *J =* 1.4 and 7.8 Hz, 1H, H-3″), 7.56 (d, *J* = 2.4 Hz, 1H, H-6), 7.65 (dd, *J* = 1.3 and 7.7 Hz, 1H, H-2″), 14.60 (s, 1H, OH); ^13^C-NMR (CDCl_3_, 100 MHz): δ 37.1, 55.4, 59.7, 110.1, 114.2, 119.9, 120.5, 124.7, 125.7, 126.7, 127.3, 130.0, 130.9, 135.3, 135.4, 136.1, 148.5, 159.5, 161.7, 172.3; HRMS (ES): MH^+^ calculated for, C_22_H_19_BrNO_2_S, 441.0028; found: 441.0021.

4-Bromo-2-((*E*)-2,3-dihydro-2-(4-isopropylphenyl)benzo[*b*][1,5]thiazepine-4-yl)phenol (**2f**)



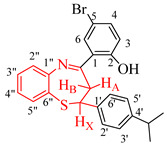



Brown solid (0.59 g, 90%), mp. 126–131 ºC; IR (ATR) ν_max_ = 464.9, 517.0, 623.4, 752.9, 825.3, 979.9, 1521.0, 1585.8, 3061.4 cm^−1^; ^1^H-NMR (CDCl_3_, 400 MHz): δ 1.27 (d, *J* = 6.9 Hz, 6H, 2 x CH_3_), 2.91 (sept, *J* = 6.9 Hz, 1H, -CH(CH_3_)_2_), 3.08 (dd, *J* = 12.0 and 13.3 Hz, 1H, H_A_), 3.31 (dd, *J* = 4.8 and 13.3 Hz, 1H, H_B_), 5.08 (dd, *J* = 4.8 and 12.0 Hz, 1H, H_X_), 6.96 (d, *J* = 8.8 Hz, 1H, H-3), 7.21 (m, 3H, H-2′.6′ and H-4″), 7.26 (m, 2H, H-3′,5′), 7.31 (dd, *J* = 1.2 and 7.9 Hz, 1H, H-4), 7.47 (dd, *J =* 2.4 and 8.4 Hz, 1H, H-5″), 7.51 (td, *J* = 1.4 and 7.8 Hz, 1H, H-3″), 7.58 (dd, *J* = 2.4 Hz, 1H, H-6), 7.66 (dd, *J* = 1.2 and 7.7 Hz, 1H, H-2″), 14.61 (s, 1H, OH); ^13^C-NMR (CDCl_3_, 100 MHz): δ 24.0, 33.9, 36.9, 60.0, 110.1, 119.9, 120.5, 124.7, 125.7, 126.1, 126.8, 127.0, 130.0, 130.9, 135.4, 136.2, 140.6, 148.4, 149.1, 161.8, 172.4; HRMS (ES): MH^+^ calculated for, C_22_H_23_BrNO_2_S, 452.0066; found: 452.0011.

### 3.4. XRD Data Collection and Refinement

Single-crystal X-ray diffraction (SC-XRD) data for **2b** were collected on a Bruker D8 Venture diffractometer with graphite-monochromated Mo κα(λ = 0.71073 Å) radiation at 173 K using an Oxford Cryostream 600 low-temperature controller. The program SAINT+ version 6.02.6 was used for data reduction and SADABS was used to make empirical absorption corrections with the space group assigned using *XPREP* [41]. The structure was solved in the *WinGX* [42] suite of programs using intrinsic phasing through *SHELXT* [43] and refined using full-matrix least-squares/difference Fourier techniques on *F*^2^ using *SHELXL-2017* [44]. All C-bound hydrogen atoms were placed at idealized positions and refined as riding atoms with isotropic parameters 1.2 or 1.5 times those of their parent atoms. Diagrams and publication material were generated using *ORTEP-3* [42] and *PLATON* [45]. Crystal data and structure refinement details for this compound are included in Table S1 in the Supplementary Materials.

### 3.5. Density Functional Theory Calculations

The density functional theory (DFT) computations were performed using the hybrid functional, composed of Becke’s three-parameter nonlocal exchange functional with the Lee–Yang–Parr correlation functional (B3LYP)2 [46], together with the LANL2DZ [47,48] basis set for all atoms. The B3LYP/6-31 + G(d,p) harmonic vibrational frequencies were calculated for the optimized geometry and also used to confirm that the stationary point is a true minimum, as no negative frequencies were found. The computations were performed in the gas phase using the Gaussian 16 software suite (Gaussian Inc., revision A.03, Wallingford, CT, USA) [49].

### 3.6. α-Glucosidase and α-Amylase Inhibition Assays of ***2a–f***

#### 3.6.1. In Vitro α-Glucosidase Inhibitory Assay of **2a**–**f**

All the tests and analyses were performed in triplicates following a literature method described in our previous study [50]. The stock solutions of the test compounds (200 µM) were prepared in DMSO, followed by dilution with a 100 mM phosphate buffer to obtain the concentrations of 100 µM. The selected assay concentrations for the test compounds (**2a**–**f**) and acarbose as a positive control were 6.25, 12.5, 25, 50 and 100 µM. The enzyme solution (0.48 u/mL α-glucosidase, 17 µL); phosphate buffer (100 mM, pH 6.8; 50 µL); and test sample in DMSO (17 µL) were incubated at 37 °C for 10 min. After pre-incubation, 17 µL of 2 mM *p*-nitrophenyl-α-d-glucopyranoside (PNP-G) was added to each of the wells containing reaction mixtures to initiate the reaction. The absorbance was measured at 405 nm using a Bio-Rad microplate reader. The IC_50_ concentration was calculated using the AAT Bioquest online calculator.

#### 3.6.2. In Vitro α-Amylase Inhibitory Assay of **2a**–**f**

The α-amylase inhibitory assay was performed in 96-well microplates using a final volume of 200 μL, following a procedure described in the literature study [51]. The enzyme assay was performed with a phosphate buffer (0.1 mM) containing 0.02% NaN_3_ and adjusted to pH 6.0 with 2.0 M phosphoric acid. The selected assay concentrations for the test compounds (**2a**–**f**) and acarbose as the positive control were 6.25, 12.5, 25, 50 and 100 µM. The enzyme solution (2.0 u/mL α-amylase, 5 µL); phosphate buffer (0.1 mM, pH 6.0; 175 µL); and 10 µL test sample were incubated at 37 °C for 10 min. After pre-incubation, 10 µL of 10 mM of 2-chloro-4-nitrophenyl-α-D-maltotrioside (CNP-G3) was added to each of the wells containing reaction mixtures to initiate the reaction. The absorbance was measured at 405 nm using a Bio-Rad microplate reader. The IC_50_ values were calculated using the AAT Bioquest online calculator.

### 3.7. Molecular Docking Studies of α-Glucosidase and α-Amylase

#### 3.7.1. Ligand Preparation

The initial structures of compounds **2a**–**f** and acarbose were generated using the Avogradro program [52]. All polar hydrogen atoms were retained. Gasteiger charges and torsional angles of the compounds were assigned using AutoDockTools [53].

#### 3.7.2. Protein Preparation, α-Glucosidase and α-Amylase

The crystal structures of α-glucosidase and α-amylase were obtained from PDB with accession number 5NN8 and 5E0F, respectively. All heteroatoms and water molecules were removed prior to the assignment of polar hydrogen atoms. Kollman–Amber united-atom partial charges and solvation parameters were added using AutoDockTools.

#### 3.7.3. Docking Simulation

Docking simulation was performed using AutoDock4.2.6 [53] with 40 × 40 × 40 grid points centred at the ligand binding site. Other parameters, such as the Lamarckian genetic algorithm, energy evaluation of 2,500,000, maximum generation of 27,000, population of 150, mutation rate of 0.02 and crossover rate of 0.8, were applied for a total of 100 docking runs for each compound. The interaction between α-glucosidase/α-amylase with all compounds was analysed using the Protein-Ligand Interaction Profiler [54].

### 3.8. Prediction of Pharmacokinetic Properties of ***2a***–***f***

The pharmacokinetic properties of compounds **2a**–**f** were predicted using Molinspiration (www.molinspiration.com). Lipinski’s rule of five [37] was used to evaluate the drug-likeness of the compounds.

### 3.9. Functional Motions of α-Glucosidase and α-Amylase in Complex with Compound 2f

The statistical structural flexibility simulation analysis on the docked complex of α-glucosidase and α-amylase with **2f** was performed using iMODS [55]. This normal mode analysis predicts the stability and flexibility of α-glucosidase and α-amylase in a complex with compound **2f**.

## 4. Conclusions

The structures and conformation of the 2-aryl-4-(4-bromo-2-hydroxyphenyl)benzo[1,5]thiazepines prepared in this study were established with the use of spectroscopic techniques and a single-crystal X-ray diffraction method. The experimental results were complemented with a computational method of **2b** as a representative example for the series. Both ^1^H-NMR and IR spectroscopic techniques indicated participation of the hydroxyl group in the intramolecular hydrogen-bonding interaction with the nitrogen atom. The presence of a six-membered intramolecularly hydrogen-bonded pseudo-aromatic ring was confirmed by single-crystal XRD and corroborated by the DFT method in the gas phase. The Hirshfeld topology analyses confirmed that the packing of these compounds is controlled, predominantly, by hydrogen bonds of the type F⋯H and Br⋯H contacts and weak H⋯H contacts. These 2-aryl-4-(4-bromo-2-hydroxyphenyl)benzo[1,5]thiazepines exhibited a moderate to increased inhibitory effect against α-glucosidase and a reduced to moderate activity against α-amylase. Derivatives **2a**, **2c** and **2f** are more likely to serve as dual inhibitors of α-glucosidase (IC_50_ = 6.70 ± 0.15, 2.69 ± 0.27 and 6.54 ± 0.11 µM, respectively) and α-amylase, (IC_50_ = 20.87 ± 0.08, 22.86 ± 0.04 and 9.71 ± 0.50 µM, respectively) and will probably help to suppress carbohydrate digestion, delay glucose uptake and result in reduced blood sugar levels with minimal gastrointestinal side effects. Compound **2f,** which exhibited increased activity against α-glucosidase (IC_50_ = 6.54 ± 0.11 µM), also exhibited a significant inhibitory effect against α-amylase (IC_50_ = 9.71 ± 0.50 µM). These preliminary in vitro enzymatic assay results and data from the molecular docking (in silico) study offer medicinal chemists an opportunity to explore further the 2,3-dihydrobenzo[*b*][1,5]thiazepine scaffold in the development of potential antidiabetic drugs and for other modes of inhibition against a variety of biochemical and biological targets. It is envisaged that cellular-based in vitro and in vivo studies, along with bioavailability and cell permeability assays, could help to clarify the mechanism of action of these benzodiazepine analogues in the body as potential multitarget agents against the pathogenesis and progression of this metabolic disorder. The presence of the Csp^2^–Br bond and the hydroxyl group on the scaffold of the test compounds, on the other hand, make these compounds suitable candidates for further chemical transformation via transition-metal-mediated cross-coupling reactions or sulphonylation to provide polycarbo-substituted or sulfonate derivatives with potential biological activity. Dehydrogenation of the C(2)–C(3) bond, on the other hand, would introduce unsaturation of the heterocyclic ring to produce planar systems.

## Figures and Tables

**Table 1:** Designation of substituent and percentage yields of compounds **2a**–**f**.

Compound	Ar	%Yield of 2
**2a**	-C_6_H_5_	85
**2b**	-C_6_H_4_(4-F)	92
**2c**	-C_6_H_4_(4-Cl)	96
**2d**	-C_6_H_4_(3-OMe)	89
**2e**	-C_6_H_4_(4-OMe)	99
**2f**	-C_6_H_4_(4-isopropyl)	90

**Table 2:** Calculated global reactivity descriptors for **2b** at B3LYP/LANL2DZ.

Parameter	2b
E_LUMO_ − E_HOMO_ (eV)	0.1395
E_LUMO +_ E_HOMO_ (eV)	0.3165
Electronegativity (χ)	−0.1583
Chemical potential (µ)	0.1583
Chemical hardness (ƞ)	0.1395
Softness (*S*)	7.692
Electrophilicity index (ω)	0.089

**Table 3:** Inhibitory effect of compounds **2a**–**f** on α-glucosidase and α-amylase.

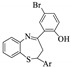		IC_50_ Values (µM ± SD) ^a^		
	Ar	α-Glucosidase	α-Amylase	SI
**2a**	-C_6_H_5_	6.70 ± 0.15	20.87 ± 0.08	0.11
**2b**	-C_6_H_4_(4-F)	17.20 ± 0.07	15.85 ± 0.05	1.09
**2c**	-C_6_H_4_(4-Cl)	2.69 ± 0.27	22.86 ± 0.04	0.12
**2d**	-C_6_H_4_(3-OMe)	13.25 ± 1.00	46.16 ± 0.31	0.29
**2e**	-C_6_H_4_(4-OMe)	15.87 ± 0.13	55.14 ± 0.19	0.29
**2f**	-C_6_H_4_(4-isopropyl)	6.54 ± 0.11	9.71 ± 0.50	0.67
Acarbose	-	7.56 ± 0.42	2.63 ± 0.22	2.76

^a^ IC_50_ values (mean ± standard error of the mean) of three independent experiments.

**Table 4:** Binding free energy estimated from docking studies for compounds **2a**–**f** and acarbose against α-glucosidase and α-amylase.

Compound	Binding Free Energy (kcal/mol)	
	α-Glucosidase	α-Amylase
**2a**	−6.48	−8.64
**2b**	−6.34	−8.54
**2c**	−7.10	−8.61
**2d**	−6.64	−9.52
**2e**	−6.76	−8.54
**2f**	−7.93	−8.78
Acarbose	−3.58	−6.72

**Table 5:** Pharmacokinetic property predictions of compounds **2a**–**f**.

Property	Compound					
	2a	2b	2c	2d	2e	2f
miLogP	6.37	6.53	7.04	6.40	6.42	7.88
Topological polar surface area; TPSA (Å)	32.59	32.59	32.59	41.83	41.83	32.59
Absorption (%); 109-(0.345 x TPSA)	97.76	97.76	97.76	94.57	94.57	97.76
Number of atoms	25	26	26	27	27	28
Molecular weight	410.34	428.33	444.78	440.36	440.36	452.42
Molecular volume	316.64	321.87	330.48	342.49	342.49	366.89
Hydrogen-bond acceptor	2	2	2	3	3	2
Hydrogen-bond donor	1	1	1	1	1	3
Rotatable bonds	2	2	2	3	3	3
Lipinski’s violation	1	1	1	1	1	1

## Data Availability

The CIF file containing complete information on the studied structure of **2b** was deposited within the Cambridge Crystallographic Data Center, CCDC 2121253, and is freely available upon request from the following website: www.ccdc.cam.ac.uk/datarequest/cif or by contacting the Cambridge Crystallographic Data Centre, 12, Union Road, Cambridge CB2 1EZ, UK; fax: +44-1223-336033; email: deposit@ccdc.cam.ac.uk.

## Supplementary Materials

The following are available online at https://www.mdpi.com/article/10.3390/molecules27206935/s1, Figure S1: Copies of NMR and IR spectra of compounds **1a**–**f** and **2a**–**f**; Figure S2: 2D NOESY (a) and HMBC (b) spectra of **2d** in CDCl3 at 400 MHz; Figure S3: Percentage inhibition of α-glucosidase by compounds **2a**–**2f** and acarbose; Figure S4: Percentage inhibition of α-amylase by compounds **2a**–**2f** and acarbose; Table S1: Crystal data and structure refinement for **2b**.

## Sample Availability

Samples of the compounds are available from the authors.
