# Supplementary material for: Synthesis, Conformational Analysis and Evaluation of the 2-aryl-4-(4-bromo-2-hydroxyphenyl)benzo[1,5]thiazepines as Potential α-Glucosidase and/or α-Amylase Inhibitors
Source: Molecules. 2022 Oct 16;27(20):6935. doi: 10.3390/molecules27206935 (PMC9607343; doi:10.3390/molecules27206935)
Supplement: Supplementary file 1 [file molecules-27-06935-s001.zip › molecules-1972585-supplementary.pdf]

molecules

Supplementary Information

Synthesis, Conformational Analysis and Evaluation of the 2-aryl-4-(4-bromo-2-hydroxyphenyl)benzo[1,5]thiazepines as Potential α-Glucosidase and/or α-Amylase Inhibitors

J.K. Nkoana, M.M. Maluleka, M.J. Mphahlele, R.M. Mampa, and Yee Siew Choong

Figure S1: Copies of ^{1}H-NMR, ^{13}C-NMR and IR spectra of compounds **1a–f** and **2a–f**

Figure S2: 2D NOESY and HMBC spectra of **2d** in $CDCl_3$ at 400 MHz

Figure S3: Percentage inhibition of α-glucosidase by **2a–2f** and acarbose

Figure S4: Percentage inhibition of α-amylase by **2a–2f** and acarbose

Table S1: Crystal data and structure refinement for **2b**

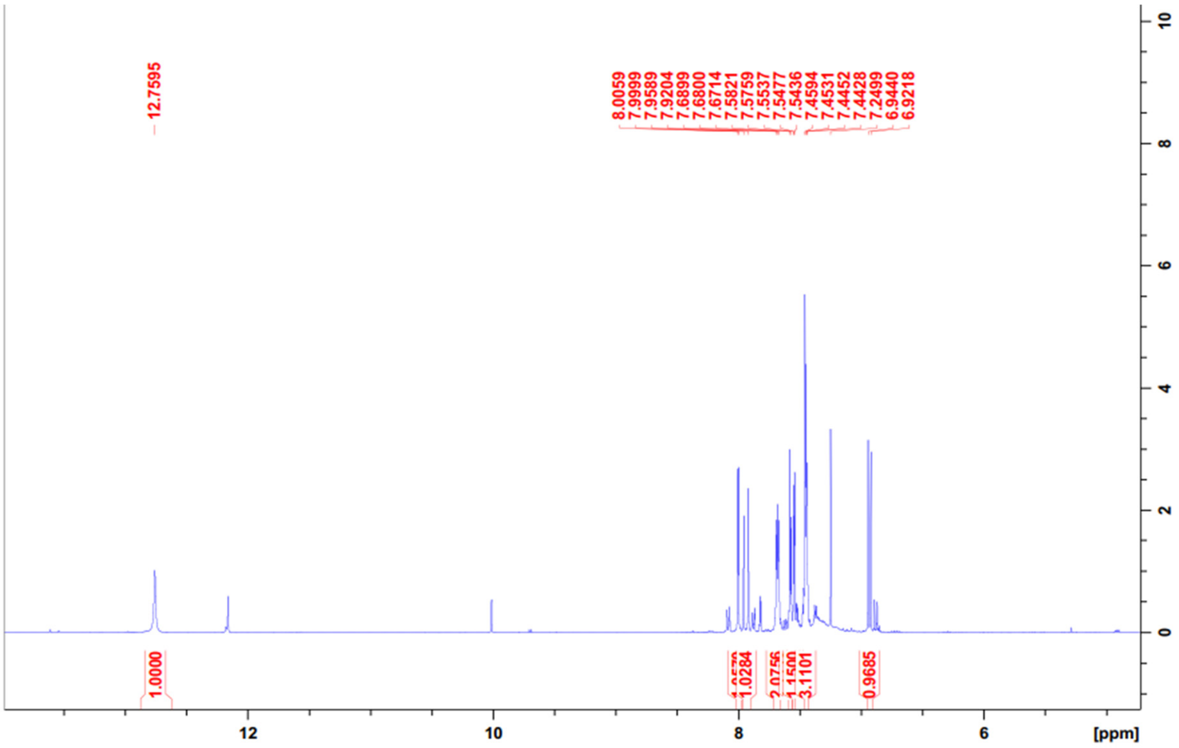
12.7595
8.0059
7.9999
7.9589
7.9204
7.6899
7.6800
7.6714
7.5821
7.5759
7.5537
7.5477
7.5436
7.4594
7.4531
7.4452
7.4428
7.2499
6.9440
6.9218
1.0000
1.0284
3.1101
0.9685
12
10
8
6
[ppm]
10
8
6
4
2
0

(a)

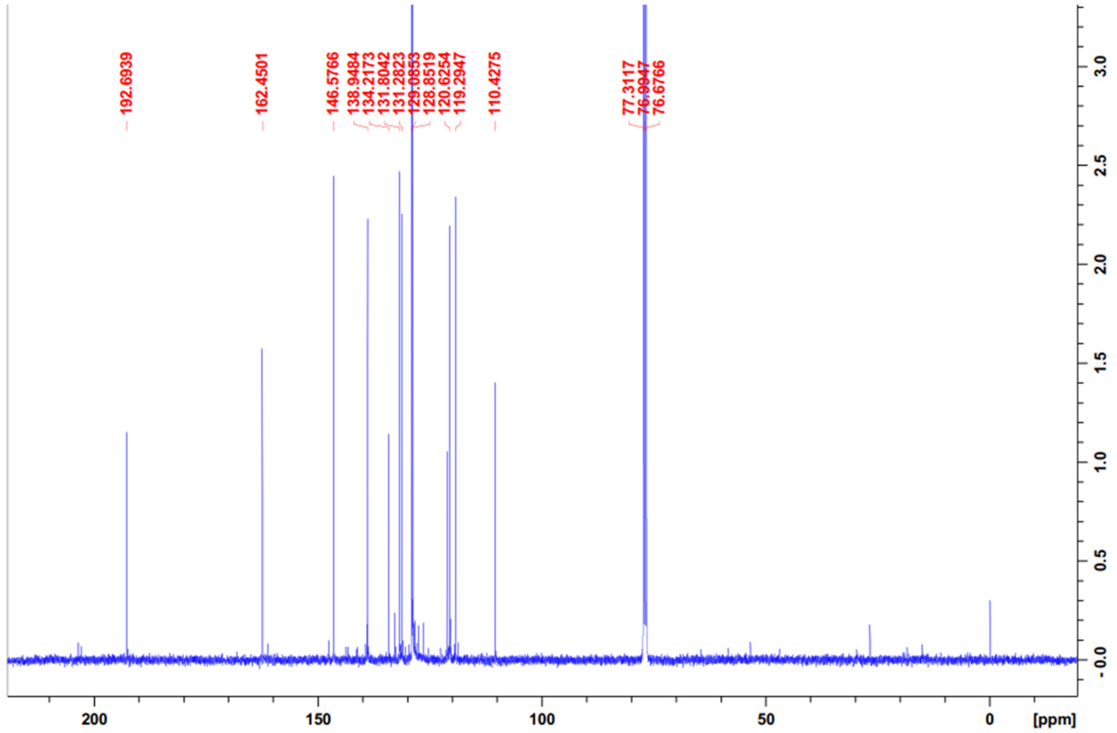
192.6939
162.4501
146.5766
138.9484
134.2173
131.8042
131.2823
129.0853
128.8519
120.6254
119.2947
110.4275
77.3117
76.9947
76.6766
200
150
100
50
0
[ppm]
3.0
2.5
2.0
1.5
1.0
0.5
-0.0

(b)

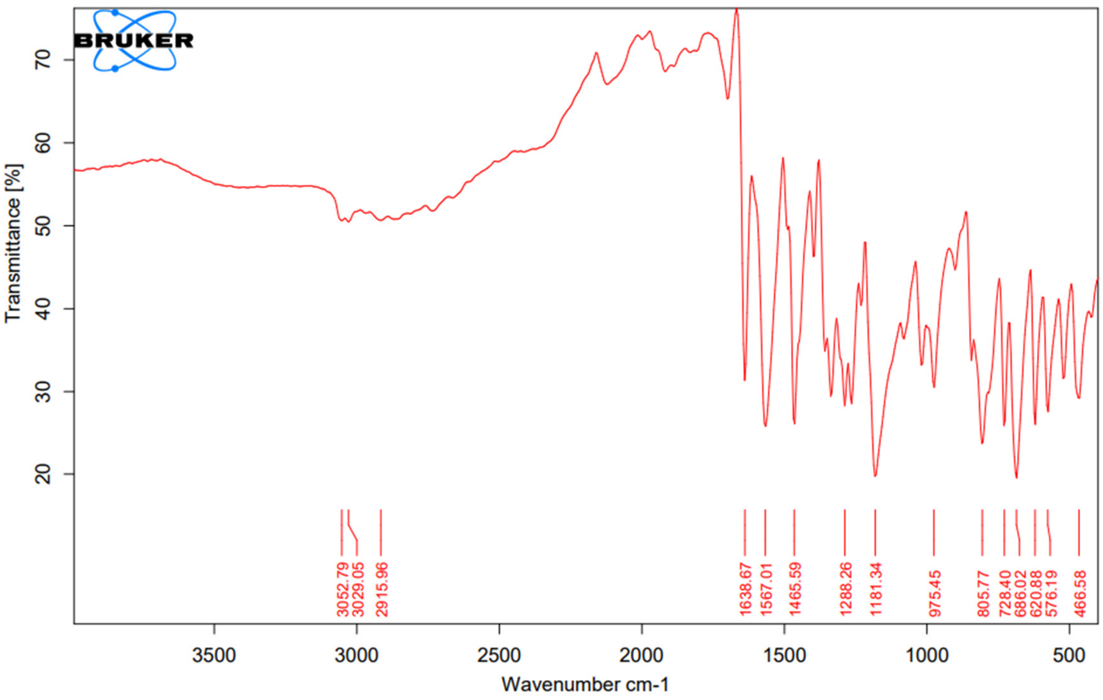

(c)

Figure S1.1. Copies of ^{1}H-NMR (a), ^{13}C-NMR (b) and IR (c) spectra of **1a**, respectively.

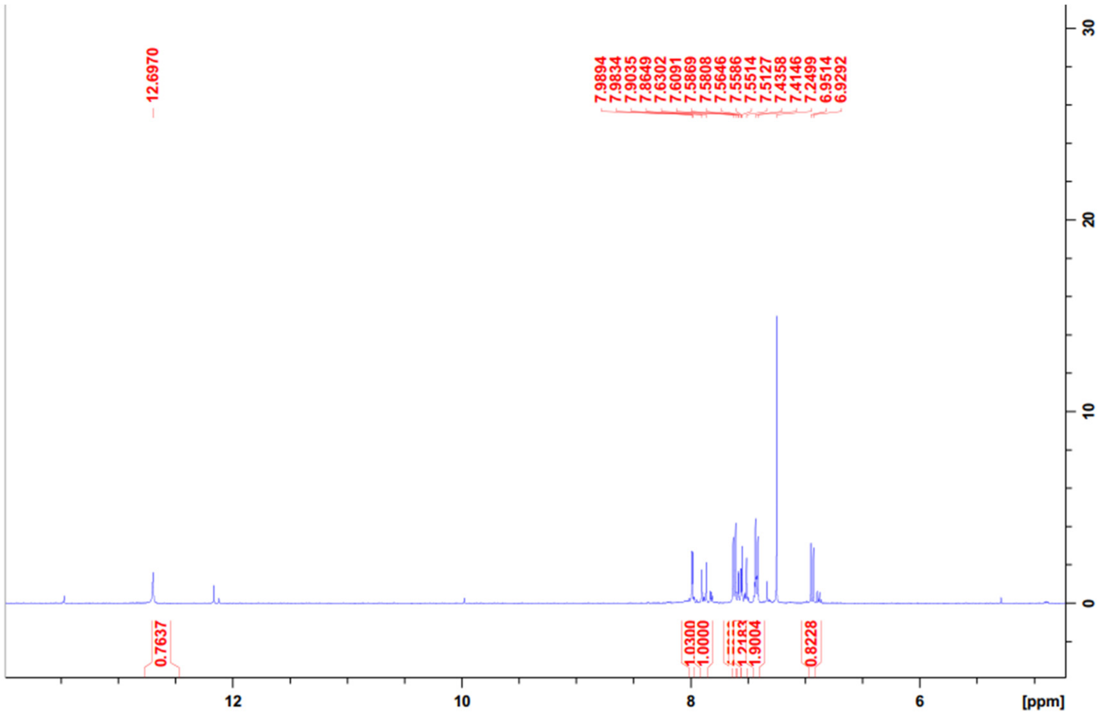

(a)

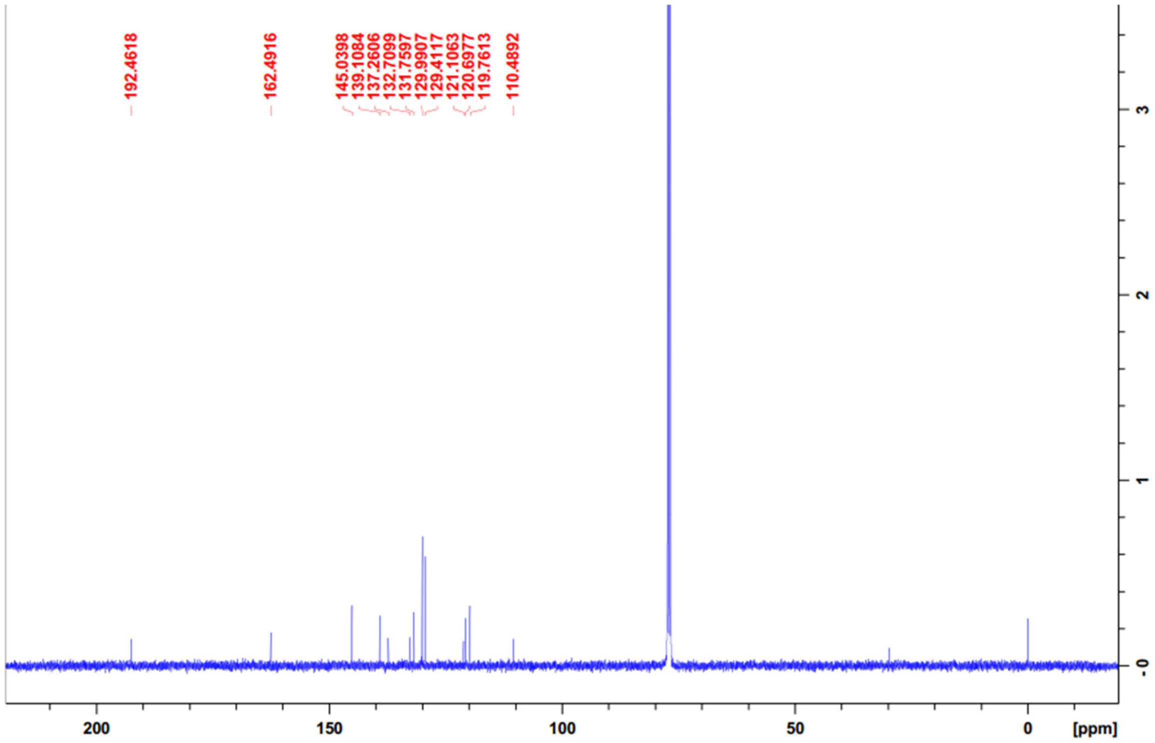

(b)

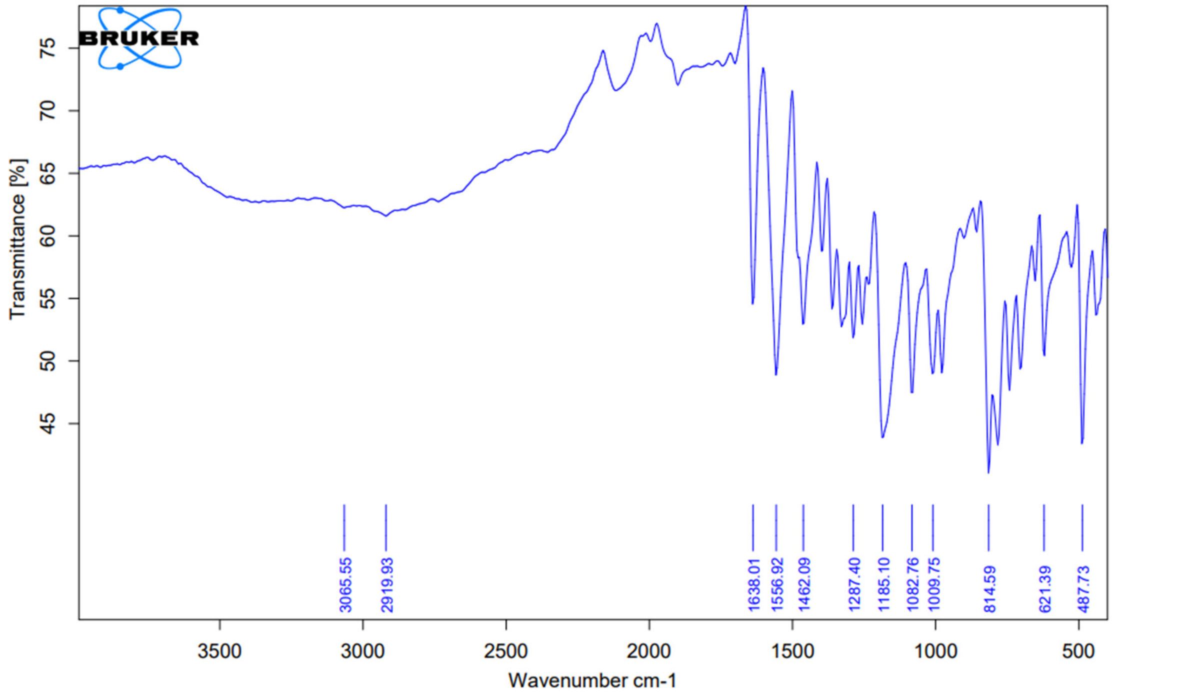

(c)

Figure S1.2. Copies of ^{1}H-NMR (a), ^{13}C-NMR (b) and IR (c) spectra of **1b**, respectively.

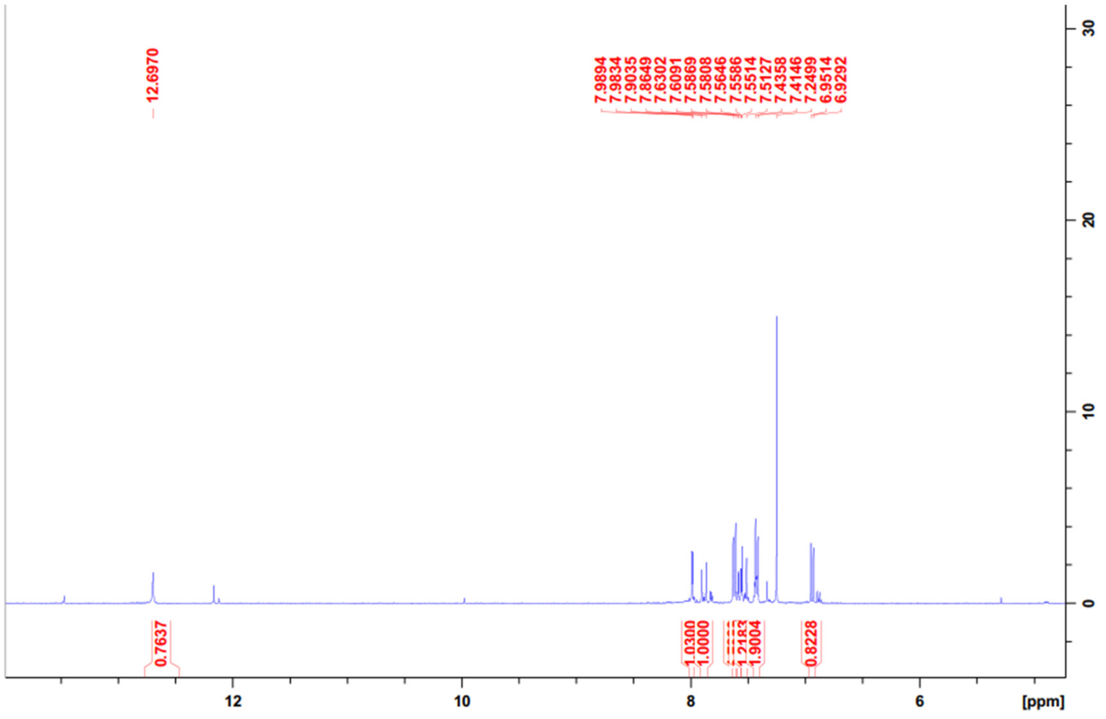
12.6970
7.9894
7.9834
7.9035
7.8649
7.6302
7.6091
7.5869
7.5808
7.5646
7.5586
7.5514
7.5127
7.4358
7.4146
7.2499
6.9514
6.9292
0.7637
1.0300
1.0000
1.2183
1.9004
0.8228
12
10
8
6
[ppm]
30
20
10
0

(a)

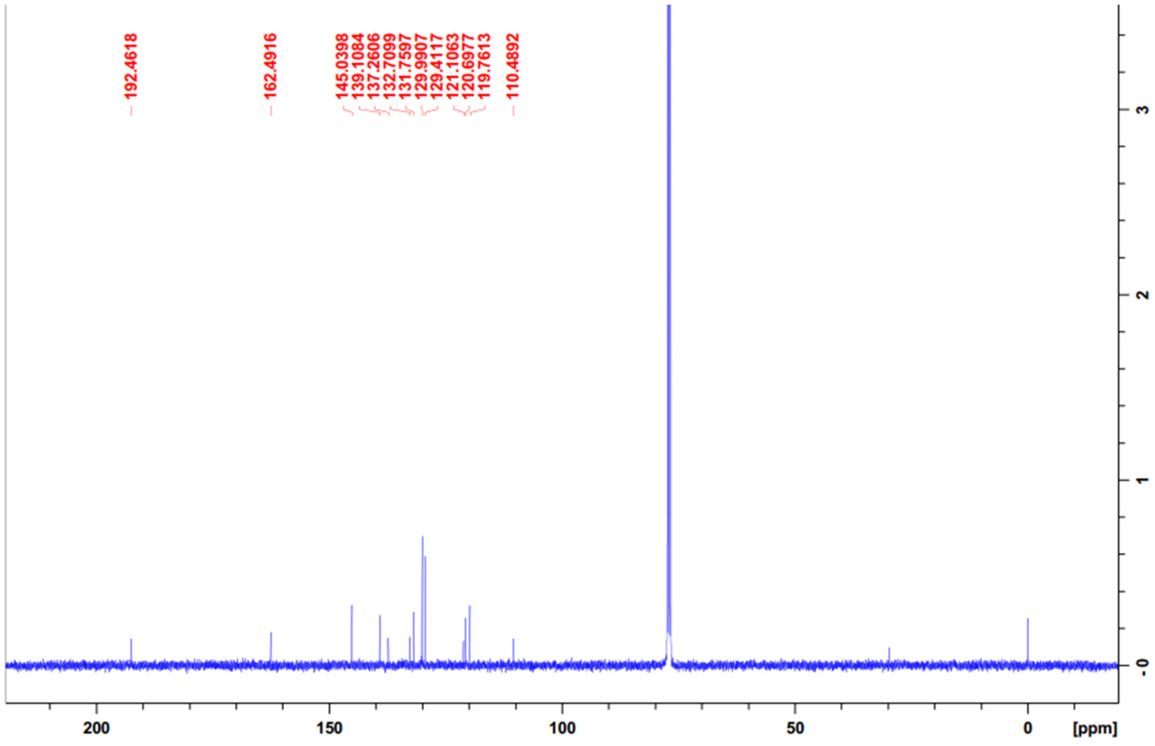
192.4618
162.4916
145.0398
139.1084
137.2606
132.7099
131.7597
129.9907
129.4117
121.1063
120.6977
119.7613
110.4892
200
150
100
50
0
[ppm]
3
2
1
-0

(b)

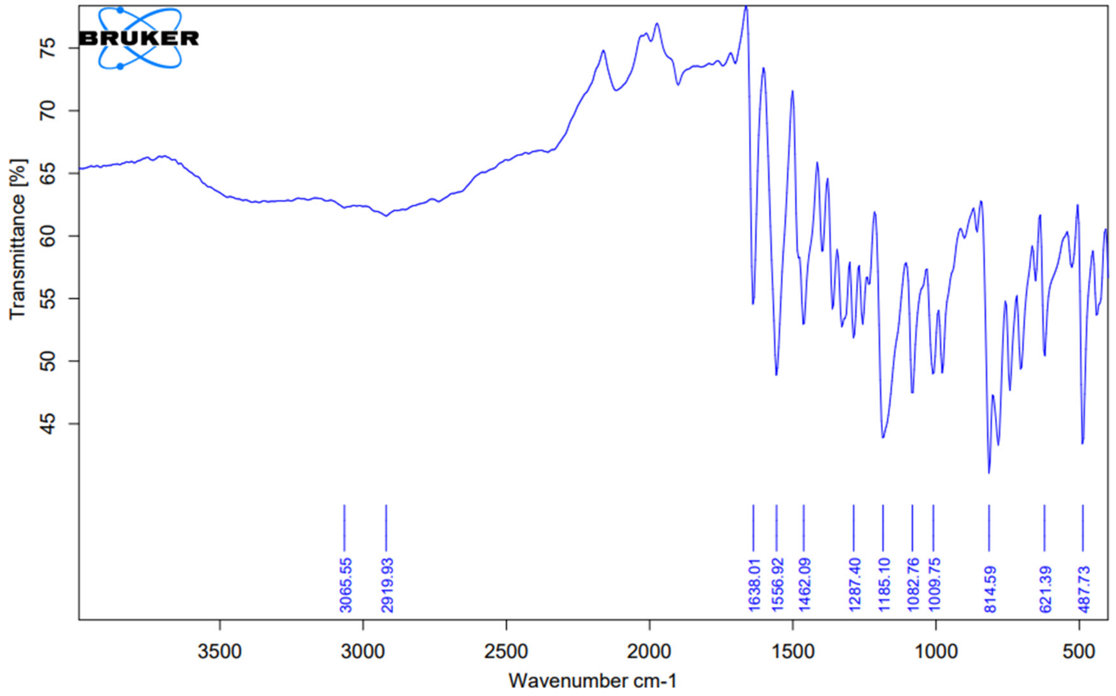

(c)

Figure S1.3. Copies of ¹H-NMR (a), ¹³C-NMR (b) and IR (c) spectra of **2c**, respectively.

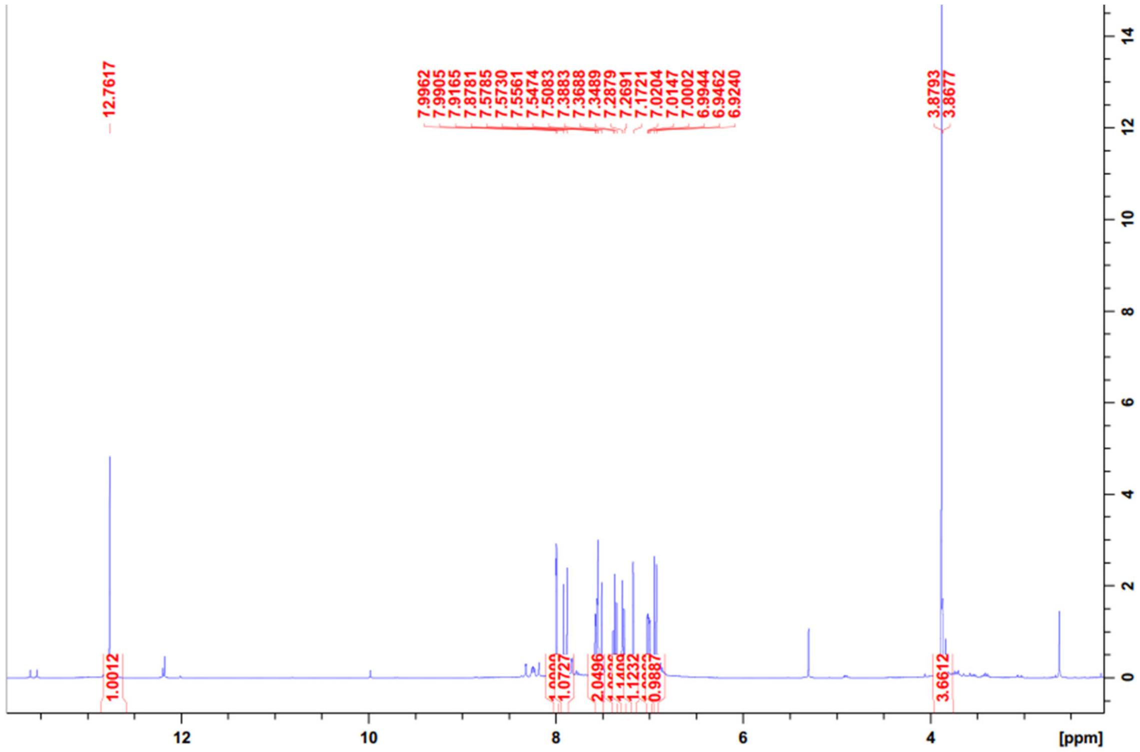

(a)

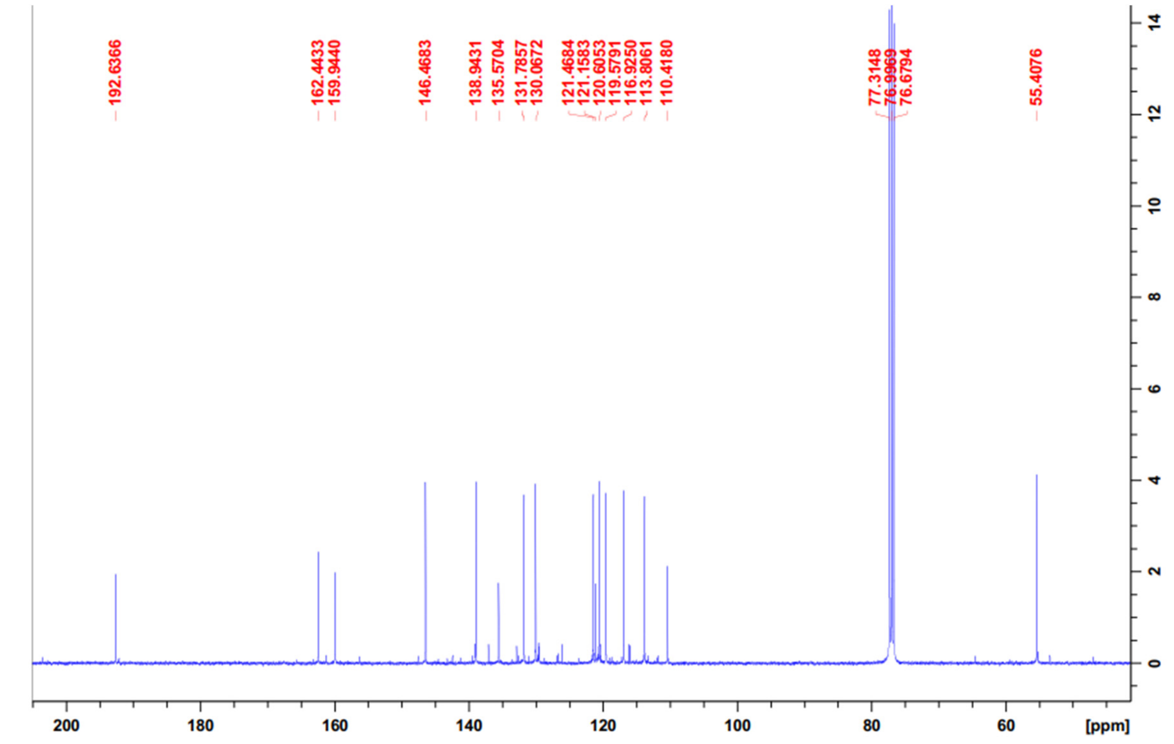

(b)

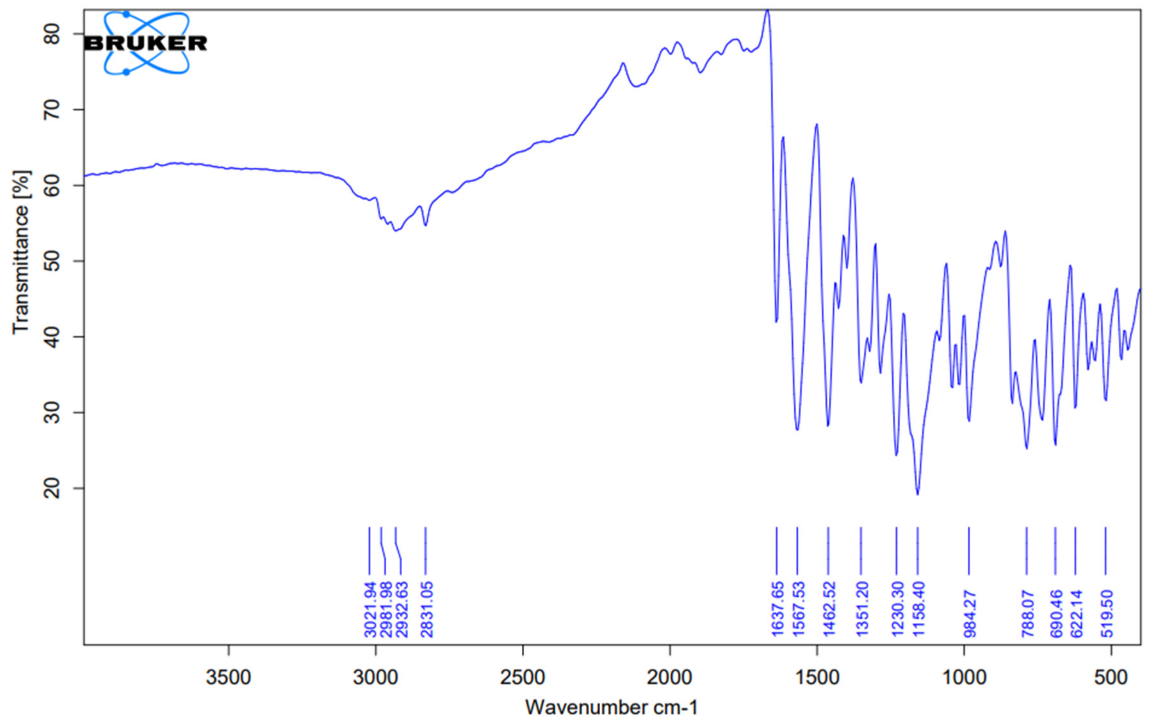

(c)

Figure S1.4. Copies of ^{1}H-NMR (a), ^{13}C-NMR (b) and IR (c) spectra of **1d**, respectively.

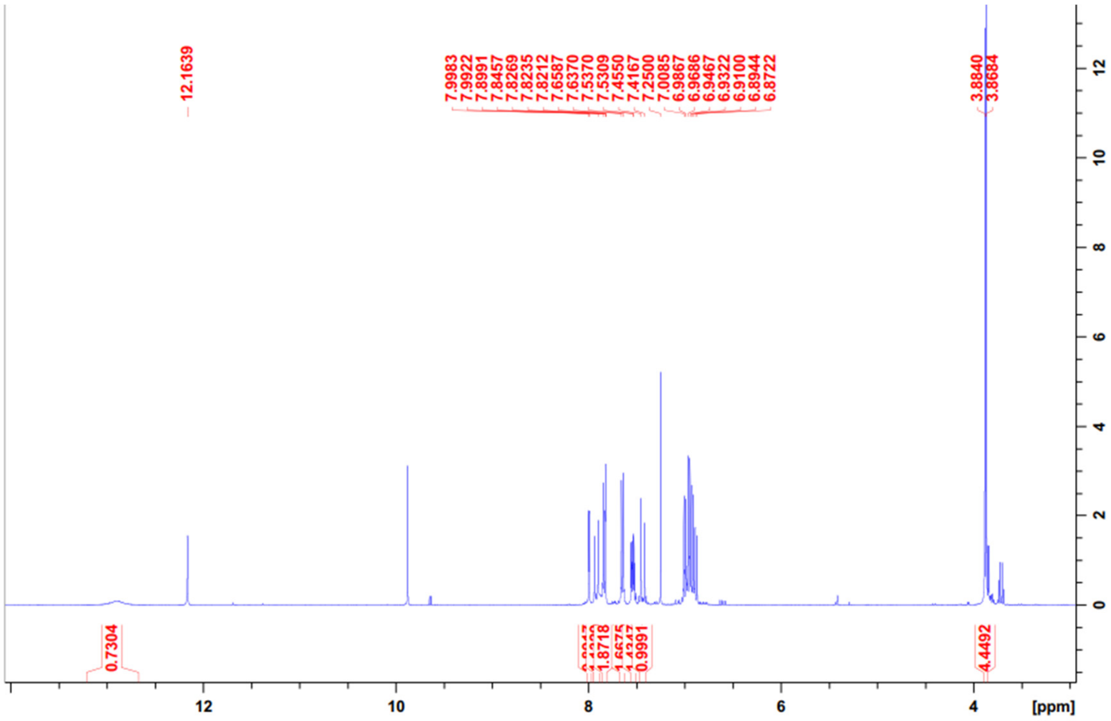
12.1639
7.9983
7.9922
7.8991
7.8457
7.8269
7.8235
7.8212
7.6587
7.6370
7.5370
7.5309
7.4550
7.4167
7.2500
7.0085
6.9867
6.9686
6.9467
6.9322
6.9100
6.8944
6.8722
3.8840
3.8684
0.7304
1.8718
0.9991
4.4492
12
10
8
6
4
[ppm]

(a)

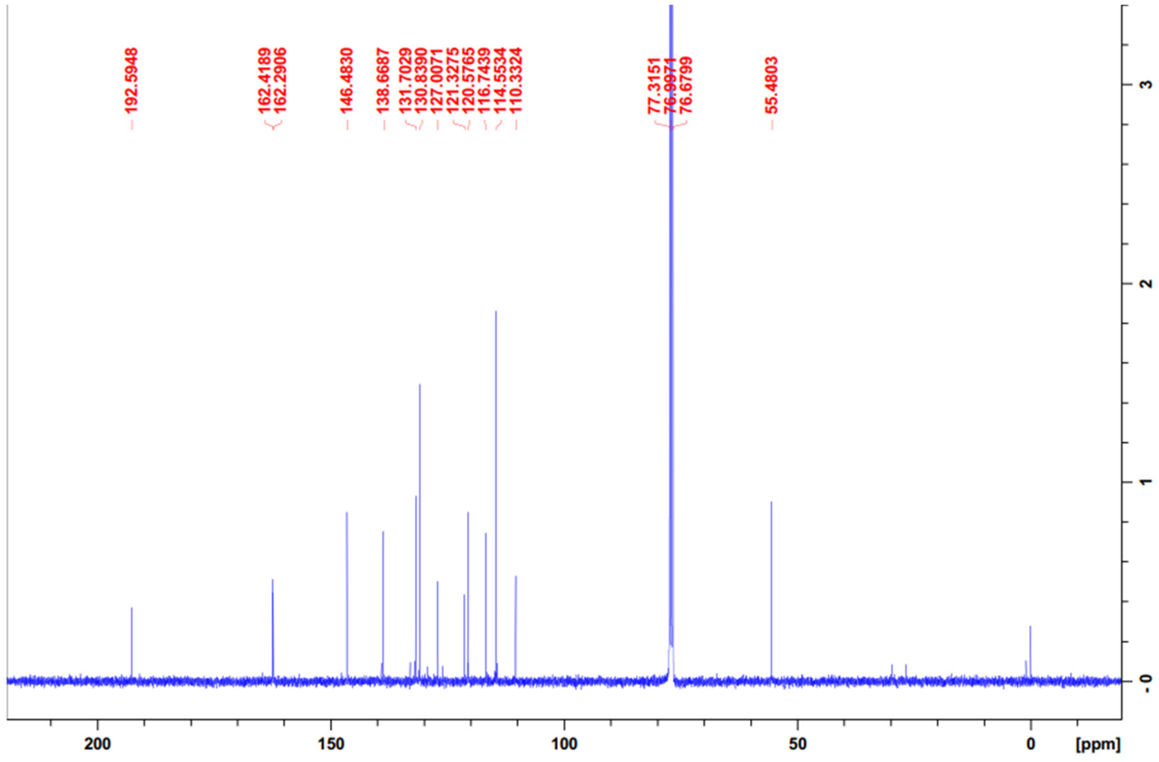
192.5948
162.4189
162.2906
146.4830
138.6687
131.7029
130.8390
127.0071
121.3275
120.5765
116.7439
114.5534
110.3324
77.3151
76.6799
55.4803
200
150
100
50
0
[ppm]

(b)

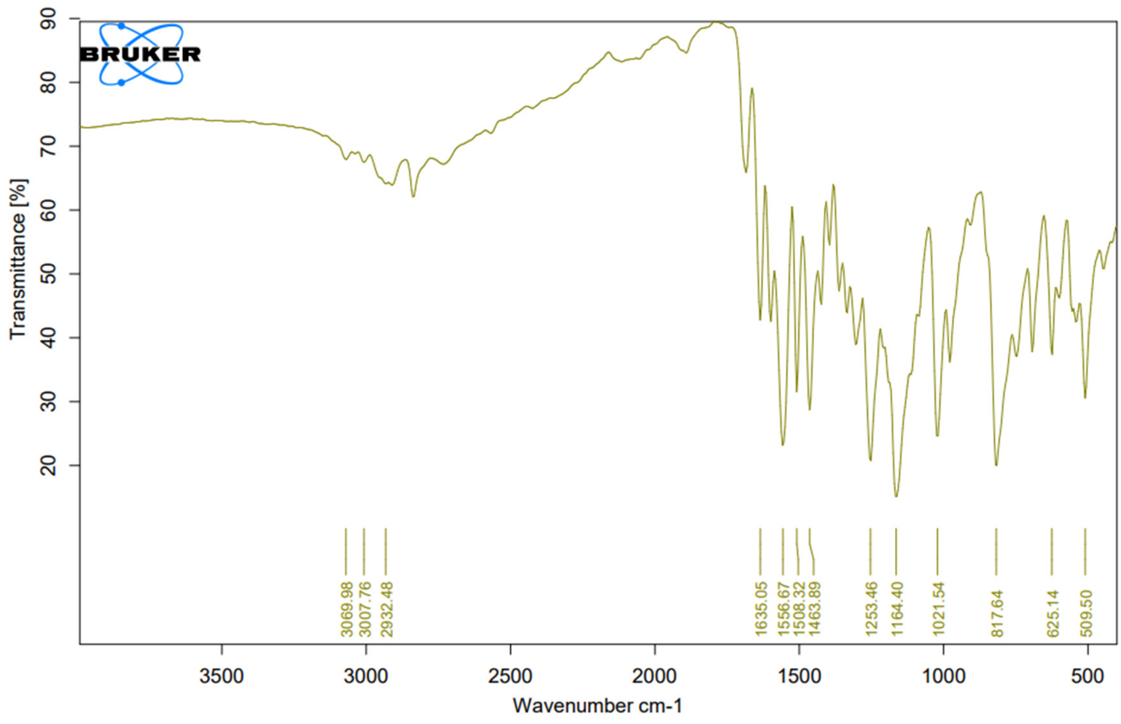

(c)

Figure S1.5. Copies of ^{1}H-NMR (a), ^{13}C-NMR (b) and IR (c) spectra of **1e**, respectively.

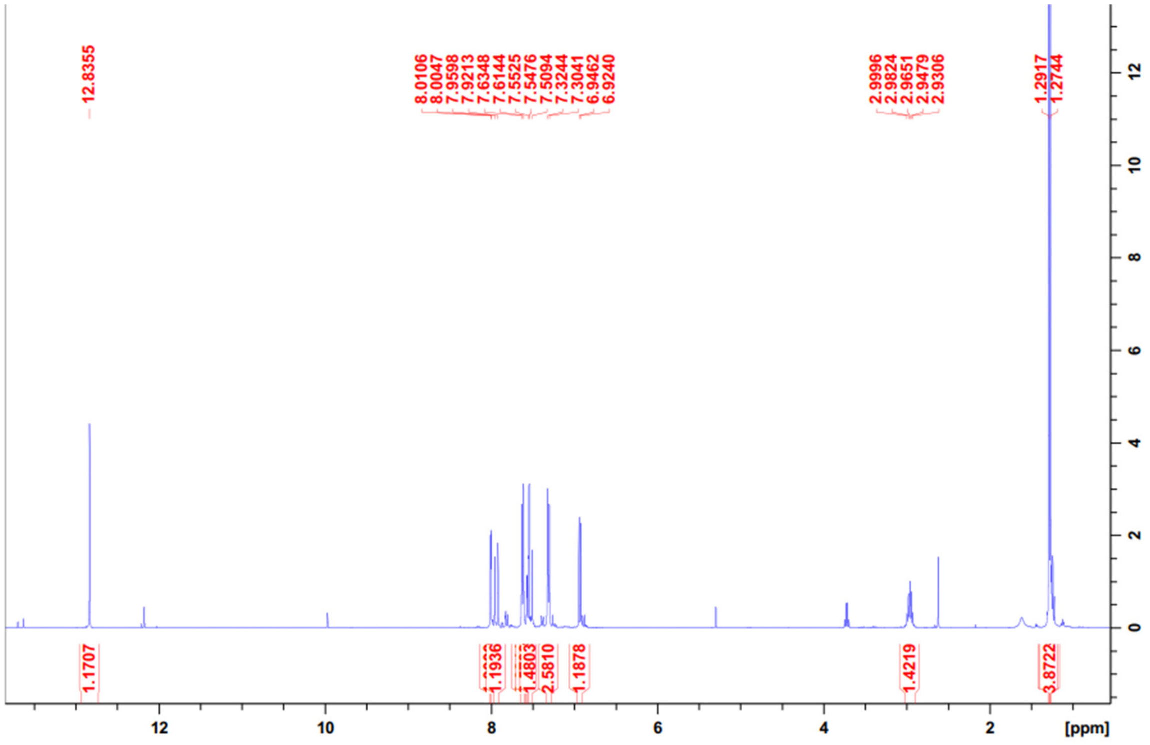

(a)

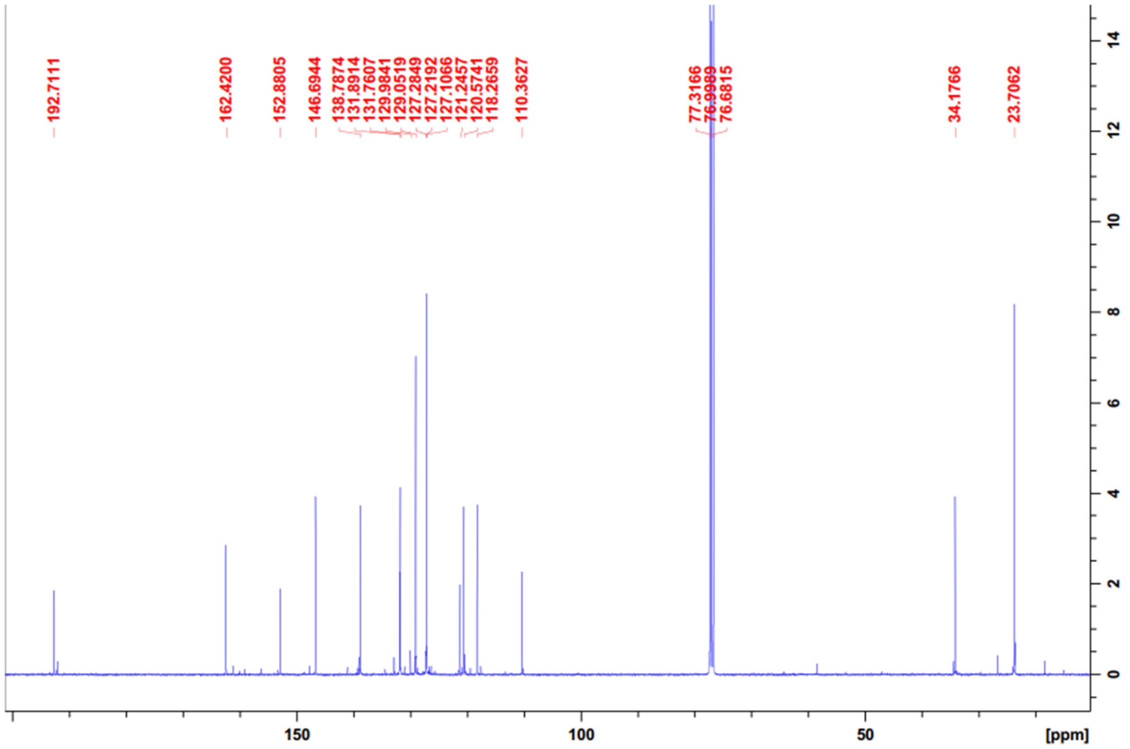

(b)

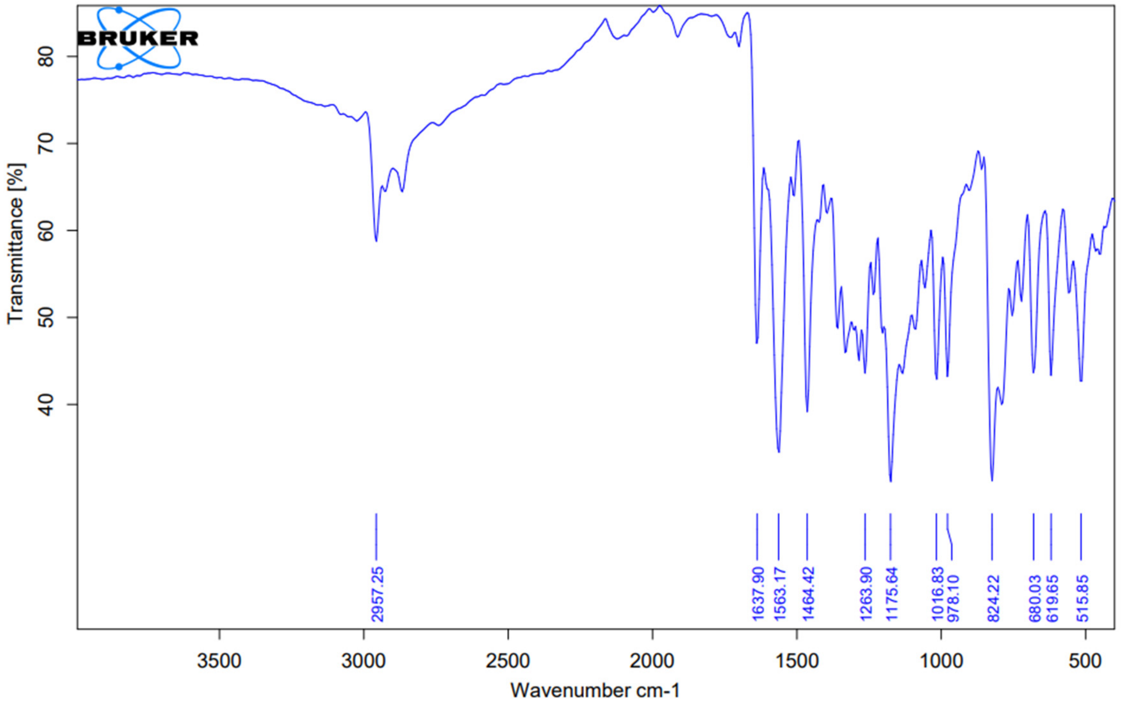

(c)

Figure S1.6. Copies of ^{1}H-NMR (a), ^{13}C-NMR (b) and IR (c) spectra of **1f**, respectively.

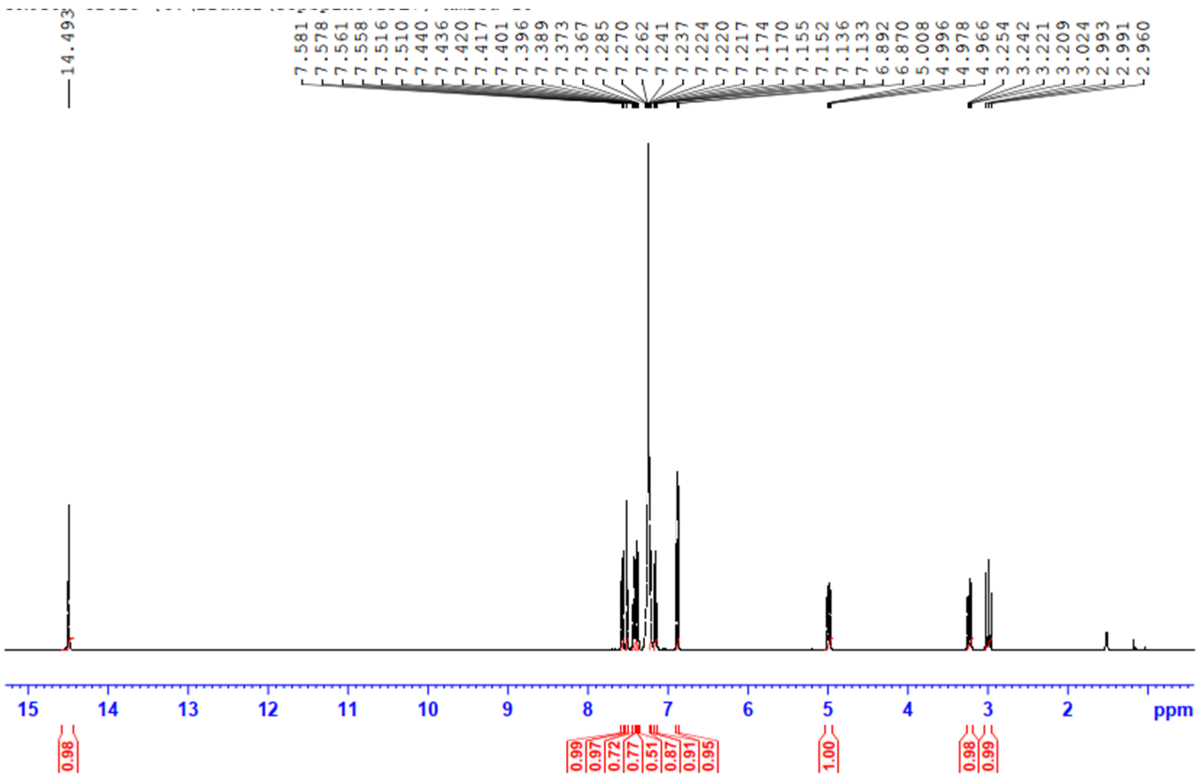
14.493
7.581
7.578
7.561
7.558
7.516
7.510
7.440
7.436
7.420
7.417
7.401
7.396
7.389
7.373
7.367
7.285
7.270
7.262
7.241
7.237
7.224
7.220
7.217
7.174
7.170
7.155
7.152
7.136
7.133
6.892
6.870
5.008
4.996
4.978
4.966
3.254
3.242
3.221
3.209
3.024
2.993
2.991
2.960
15 14 13 12 11 10 9 8 7 6 5 4 3 2 ppm
0.98
0.99 0.97 0.72 0.77 0.51 0.87 0.91 0.95
1.00
0.98 0.99

(a)

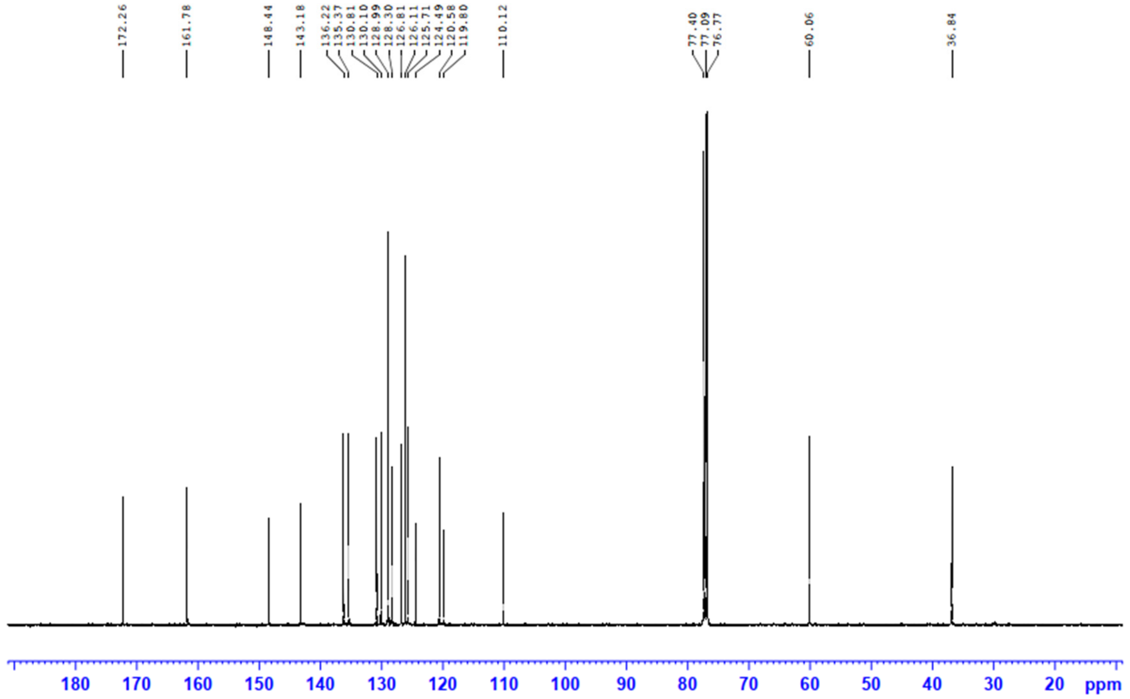
172.26
161.78
148.44
143.18
136.22
135.37
130.81
130.10
128.99
128.30
126.81
126.11
125.71
124.49
120.58
119.80
110.12
77.40
77.09
76.77
60.06
36.84
180 170 160 150 140 130 120 110 100 90 80 70 60 50 40 30 20 ppm

(b)

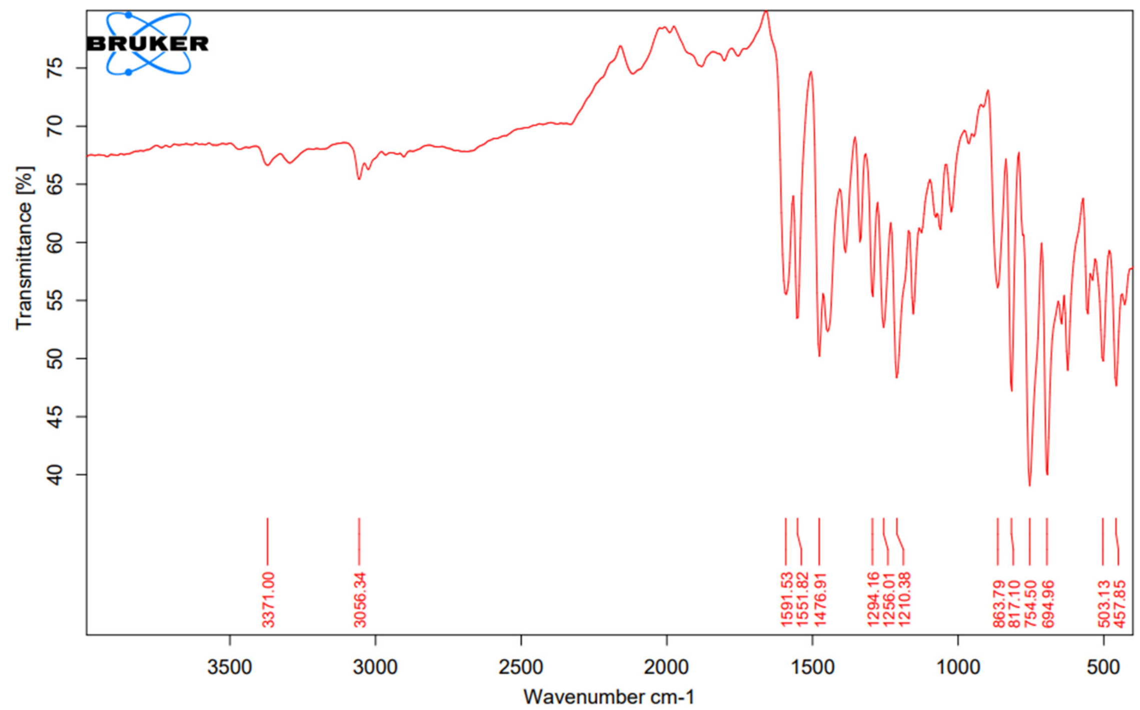

(c)

Figure S1.7. Copies of ¹H-NMR (a), ¹³C-NMR (b) and IR (c) spectra of **2a**, respectively.

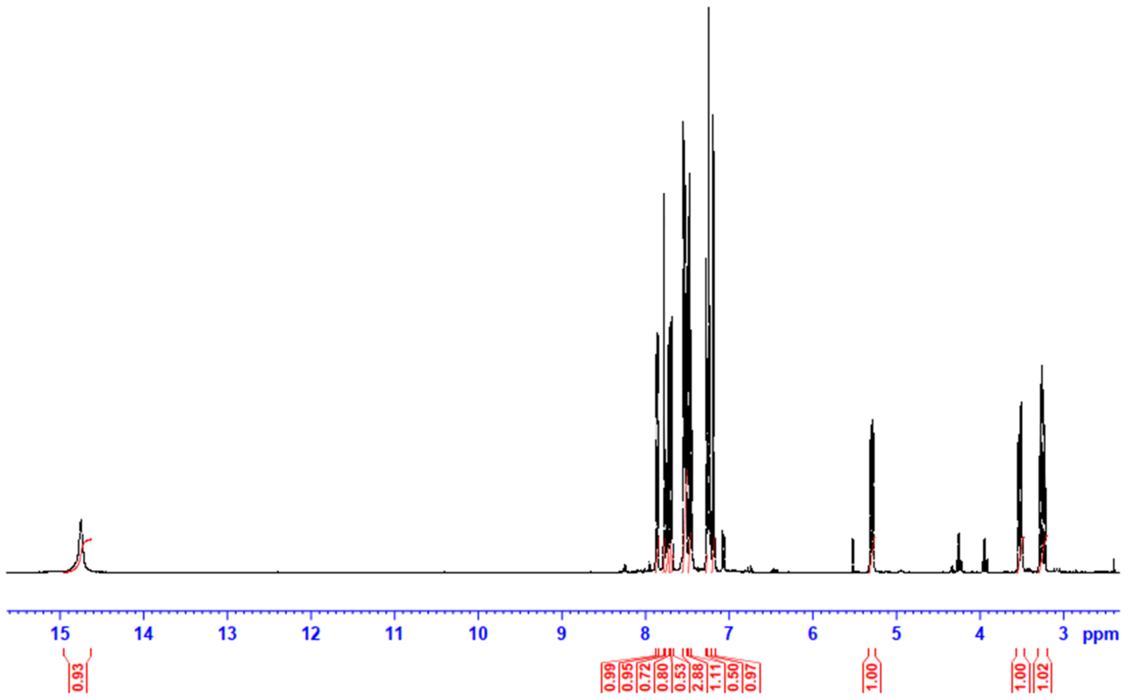

(a)

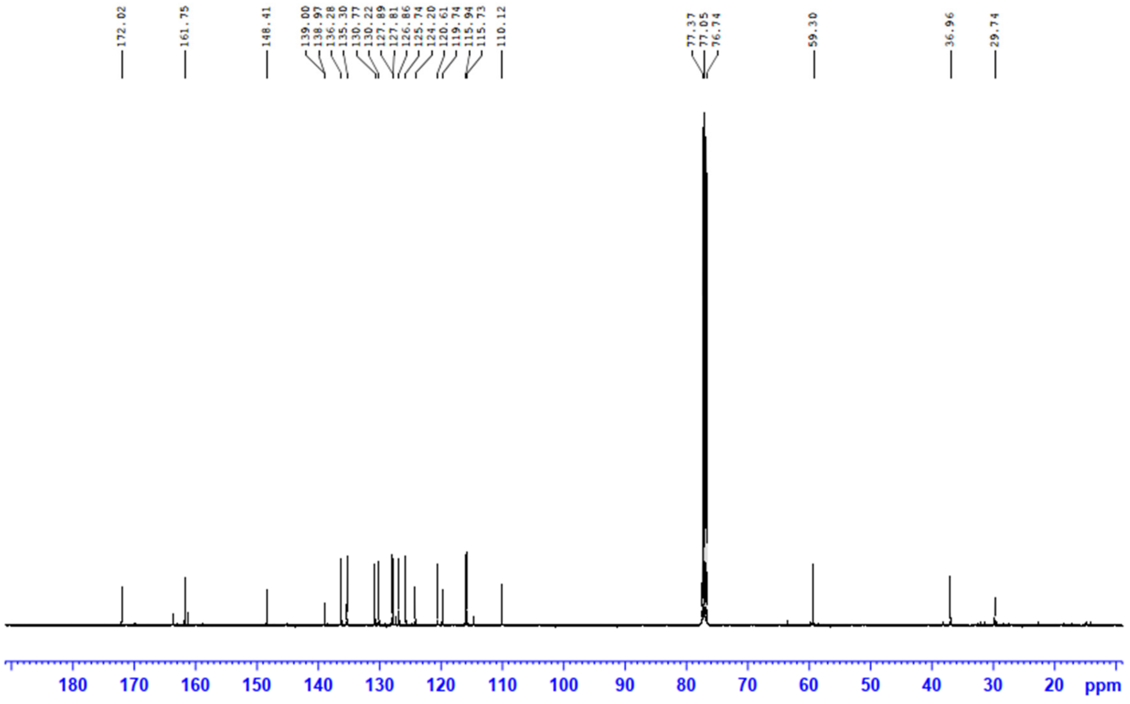

(b)

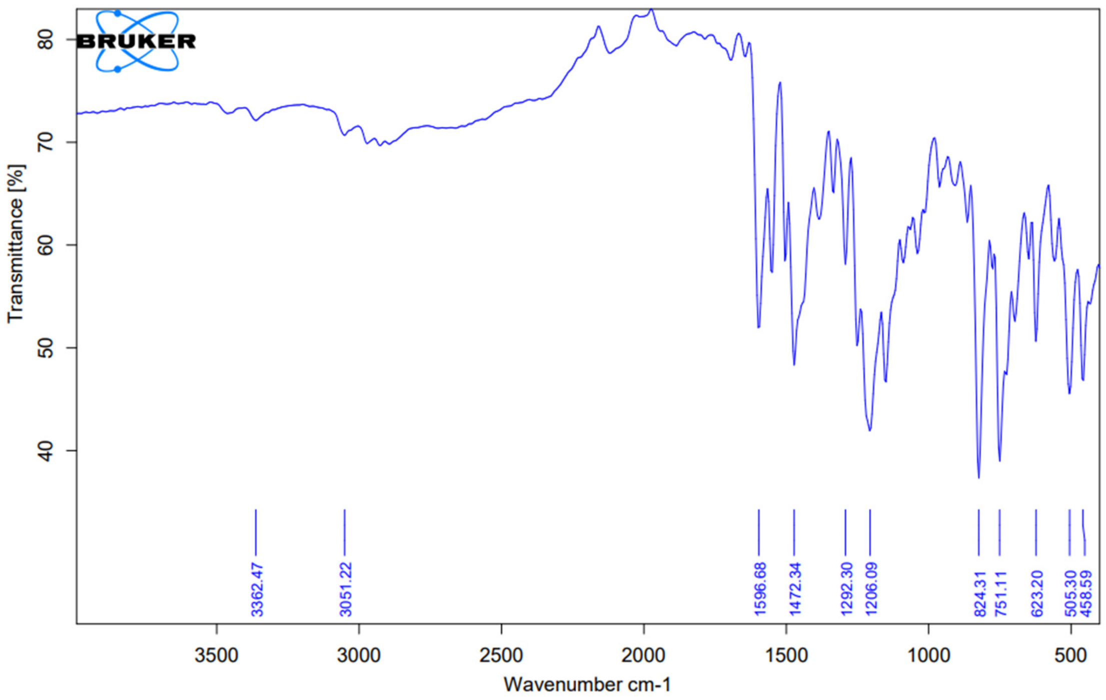

(c)

Figure S1.8. Copies of ^{1}H-NMR (a), ^{13}C-NMR (b) and IR (c) spectra of **2b**, respectively.

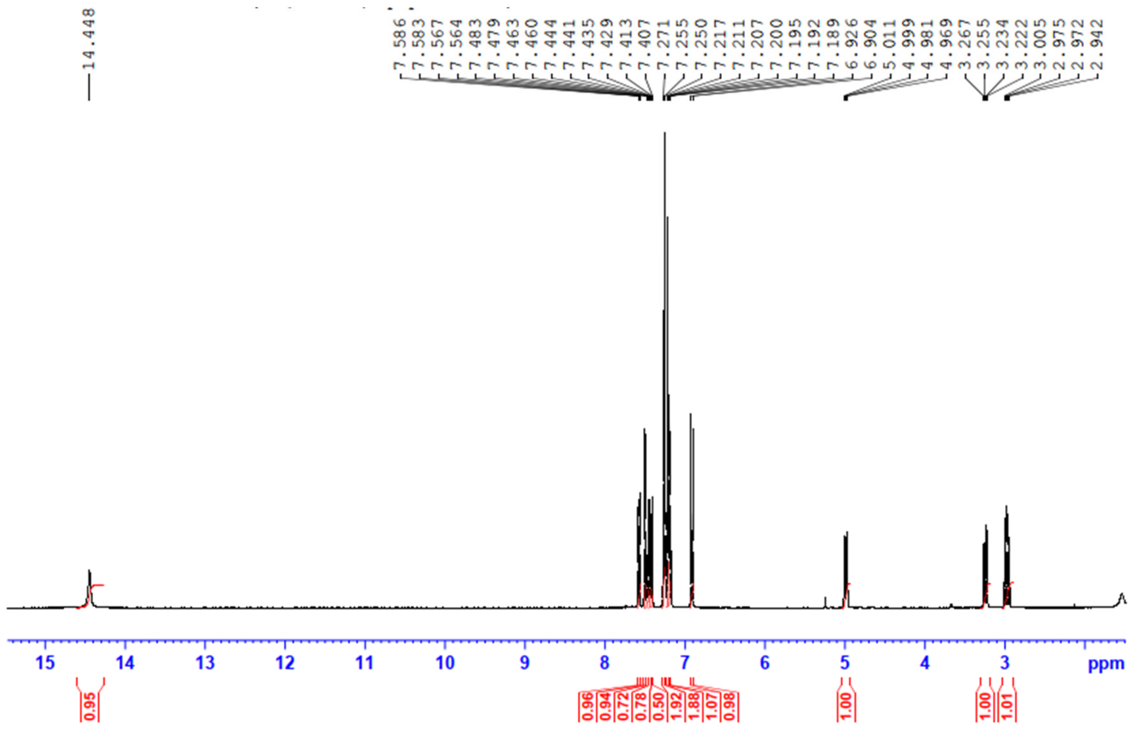
14.448
7.586
7.583
7.567
7.564
7.483
7.479
7.463
7.460
7.444
7.441
7.435
7.429
7.413
7.407
7.271
7.255
7.250
7.217
7.211
7.207
7.200
7.195
7.192
7.189
6.926
6.904
5.011
4.999
4.981
4.969
3.267
3.255
3.234
3.222
3.005
2.975
2.972
2.942
15 14 13 12 11 10 9 8 7 6 5 4 3 ppm
0.95
0.96 0.94 0.72 0.78 0.50 1.92 1.88 1.07 0.98
1.00
1.00 1.01

(a)

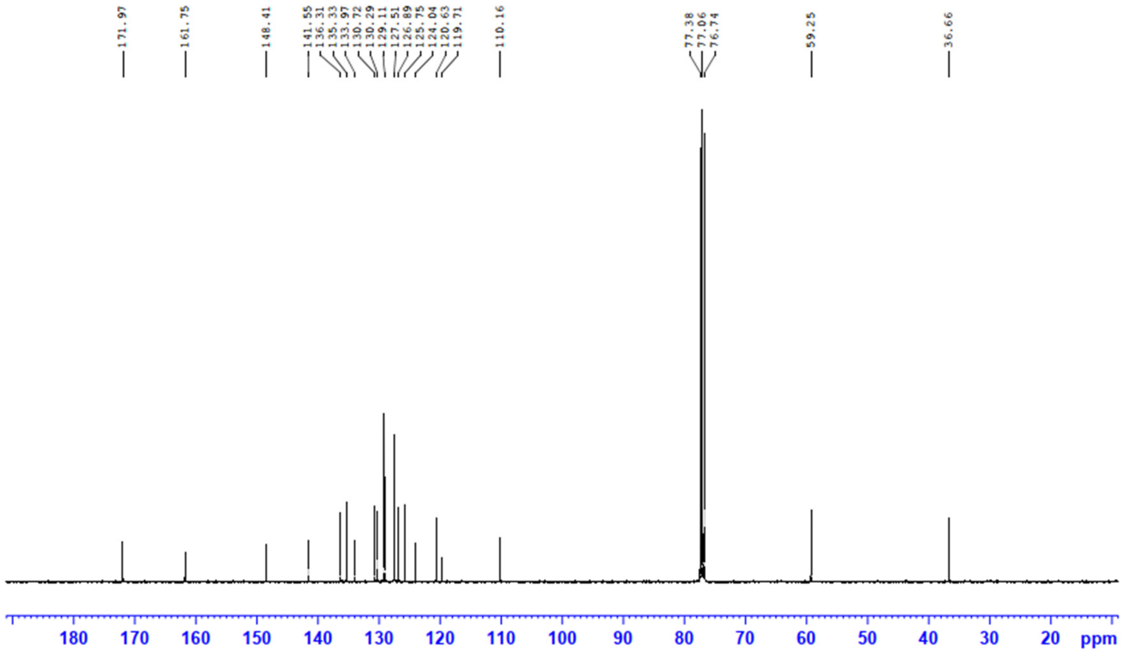
171.97
161.75
148.41
141.55
136.31
135.33
133.97
130.72
130.29
129.11
127.51
126.89
125.75
124.04
120.63
119.71
110.16
77.38
77.06
76.74
59.25
36.66
180 170 160 150 140 130 120 110 100 90 80 70 60 50 40 30 20 ppm

(b)

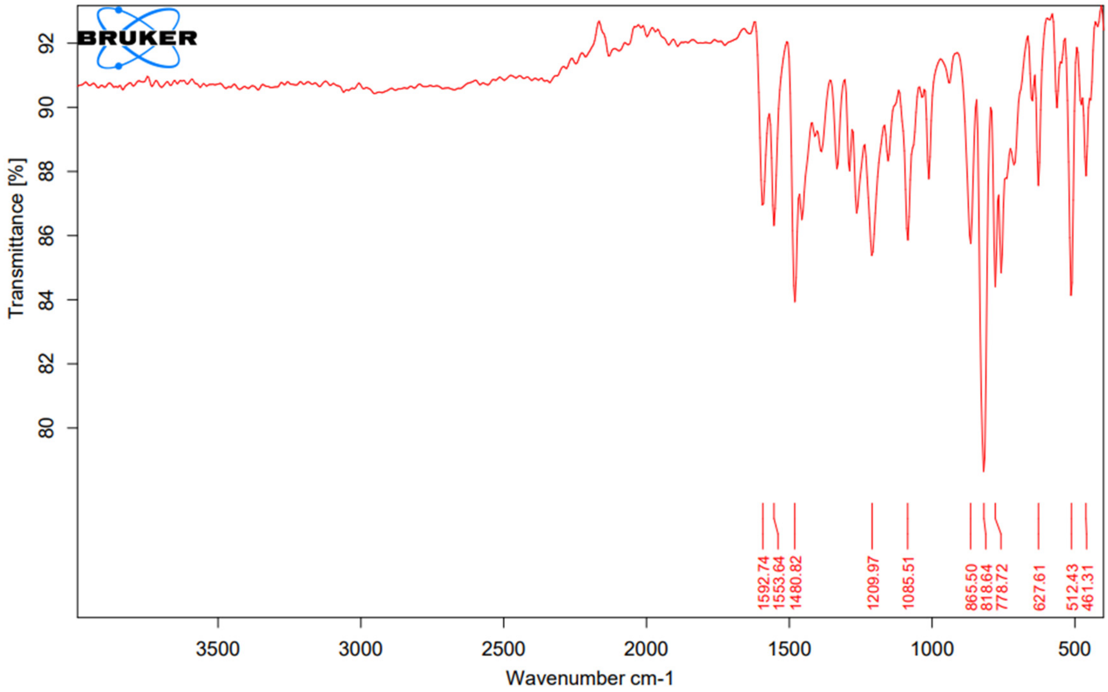

(c)

Figure S1.9. Copies of ^{1}H-NMR (a), ^{13}C-NMR (b) and IR (c) spectra of **2c**, respectively.

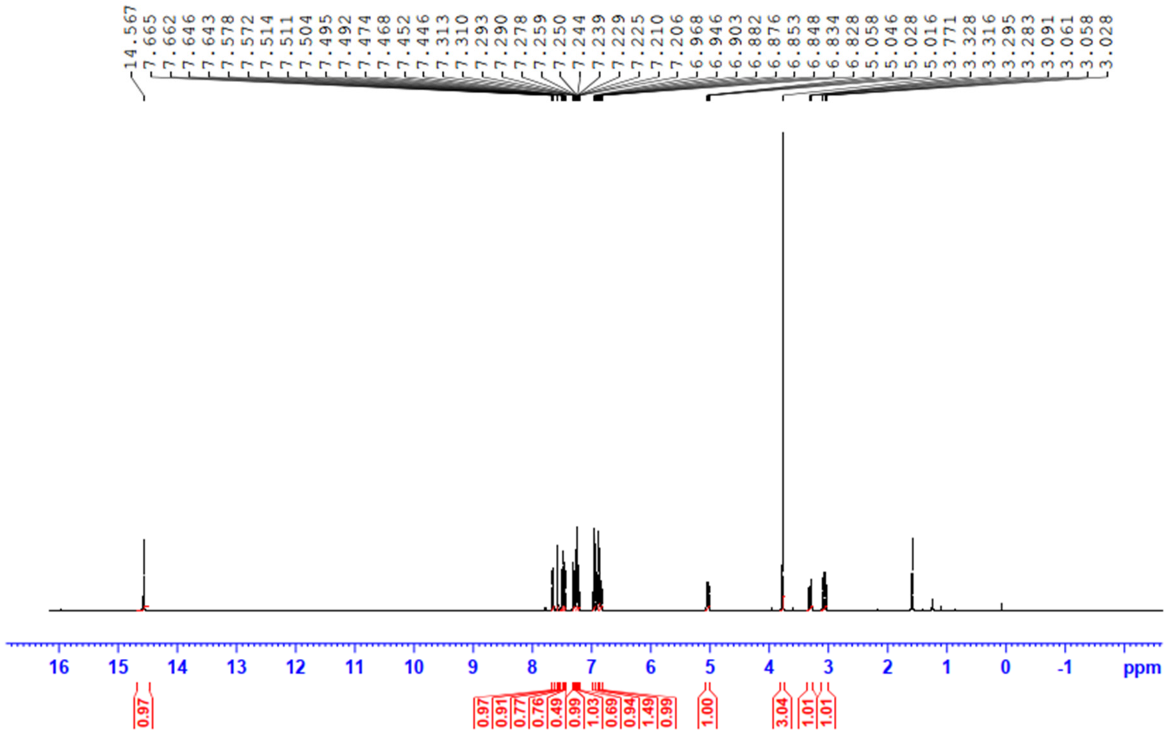

(a)

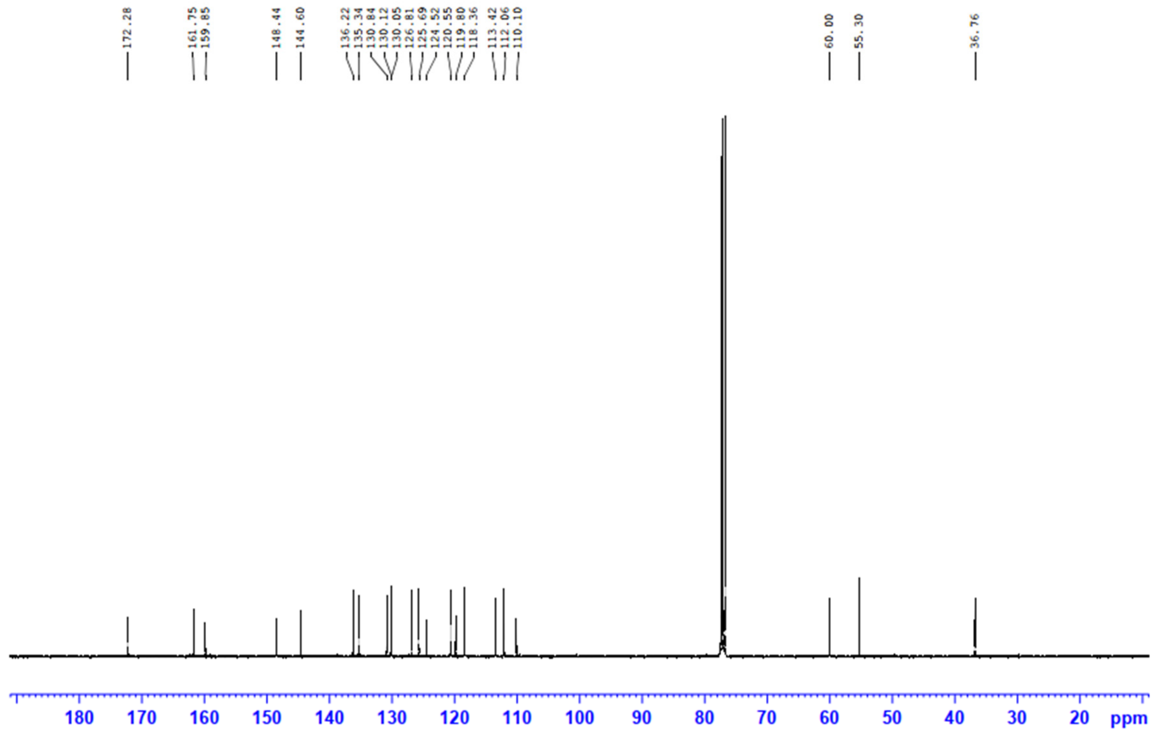

(b)

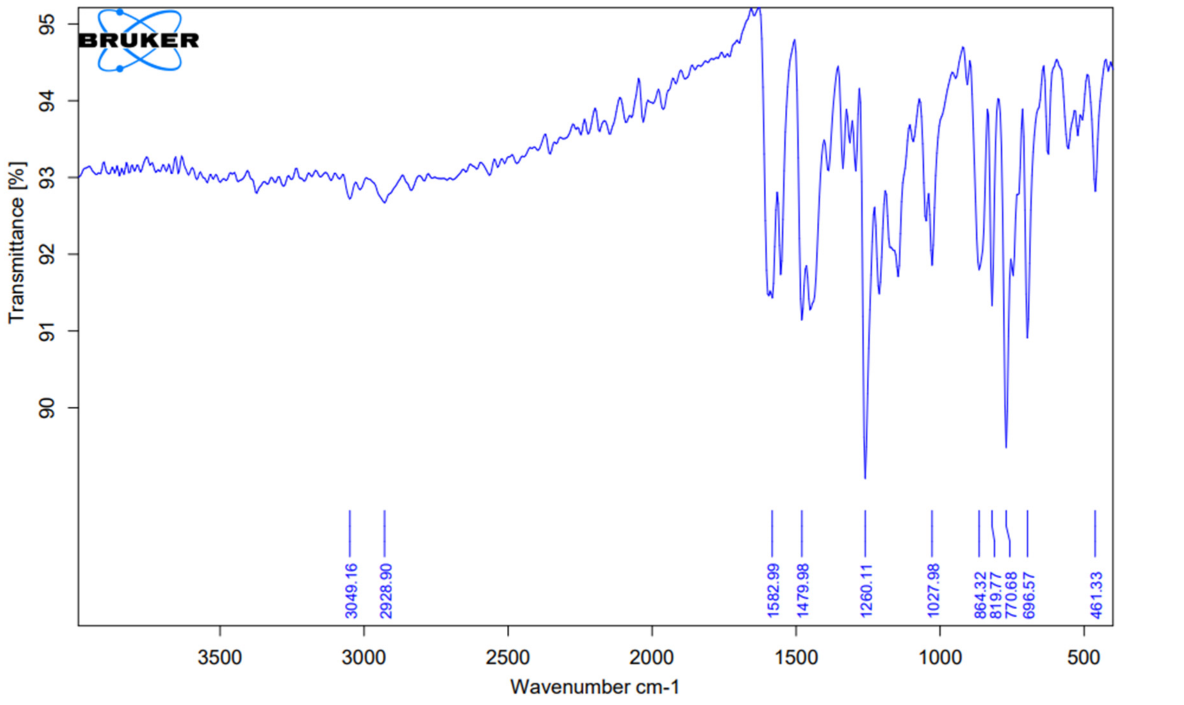

(c)

Figure S1.10. Copies of ^{1}H-NMR (a), ^{13}C-NMR (b) and IR (c) spectra of **2d**, respectively.

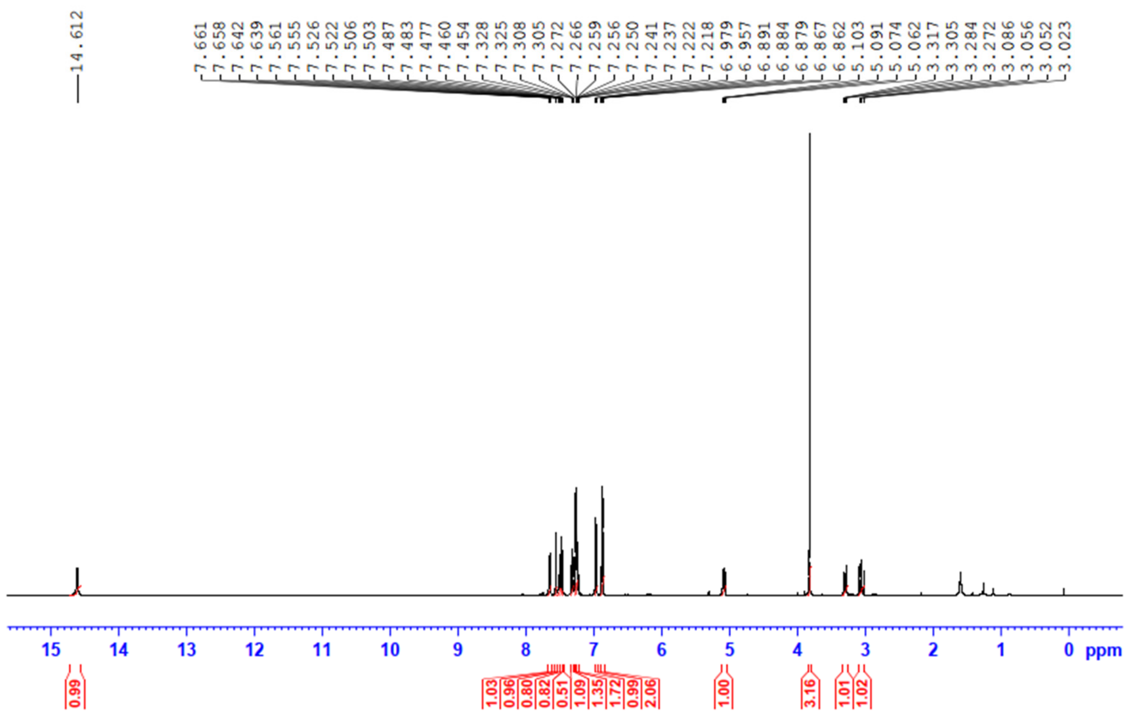
14.612
7.661
7.658
7.642
7.639
7.561
7.555
7.526
7.522
7.506
7.503
7.487
7.483
7.477
7.460
7.454
7.328
7.325
7.308
7.305
7.272
7.266
7.259
7.256
7.250
7.241
7.237
7.222
7.218
6.979
6.957
6.891
6.884
6.879
6.867
6.862
5.103
5.091
5.074
5.062
3.317
3.305
3.284
3.272
3.086
3.056
3.052
3.023
15 14 13 12 11 10 9 8 7 6 5 4 3 2 1 0 ppm
0.99
1.03
0.96
0.80
0.82
0.51
1.09
1.35
1.72
0.99
2.06
1.00
3.16
1.01
1.02
(a)

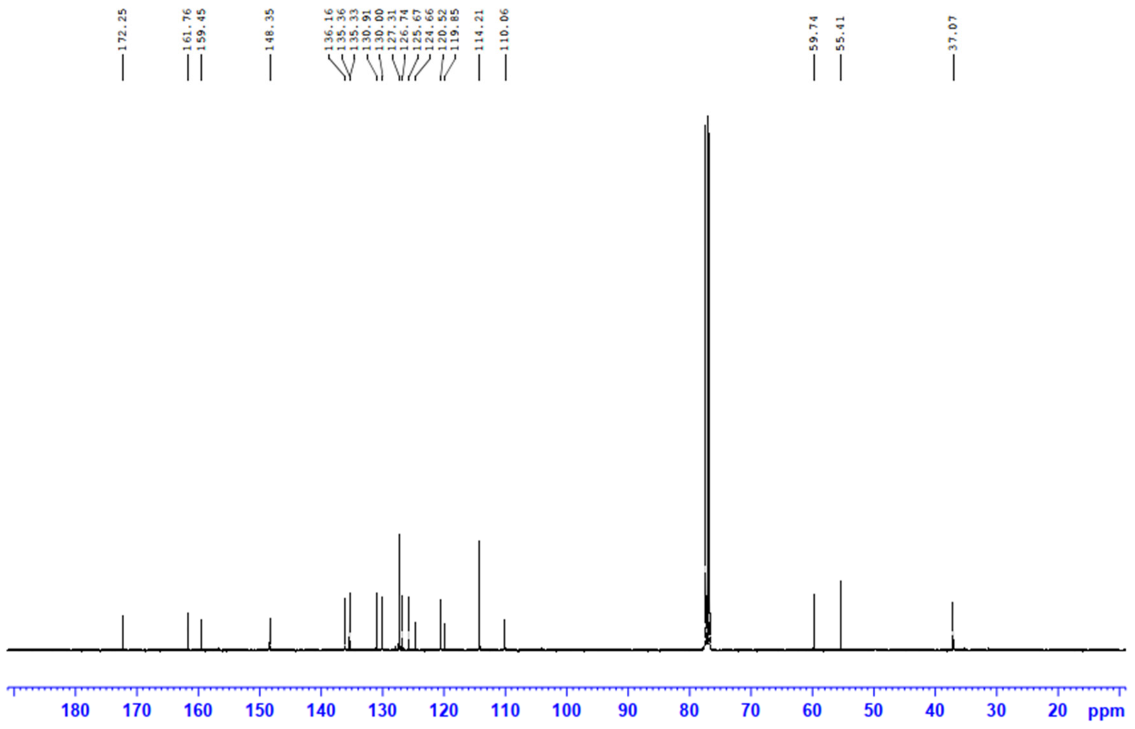
172.25
161.76
159.45
148.35
136.16
135.36
135.33
130.91
130.00
127.31
126.74
125.67
124.66
120.52
119.85
114.21
110.06
59.74
55.41
37.07
180 170 160 150 140 130 120 110 100 90 80 70 60 50 40 30 20 ppm
(b)

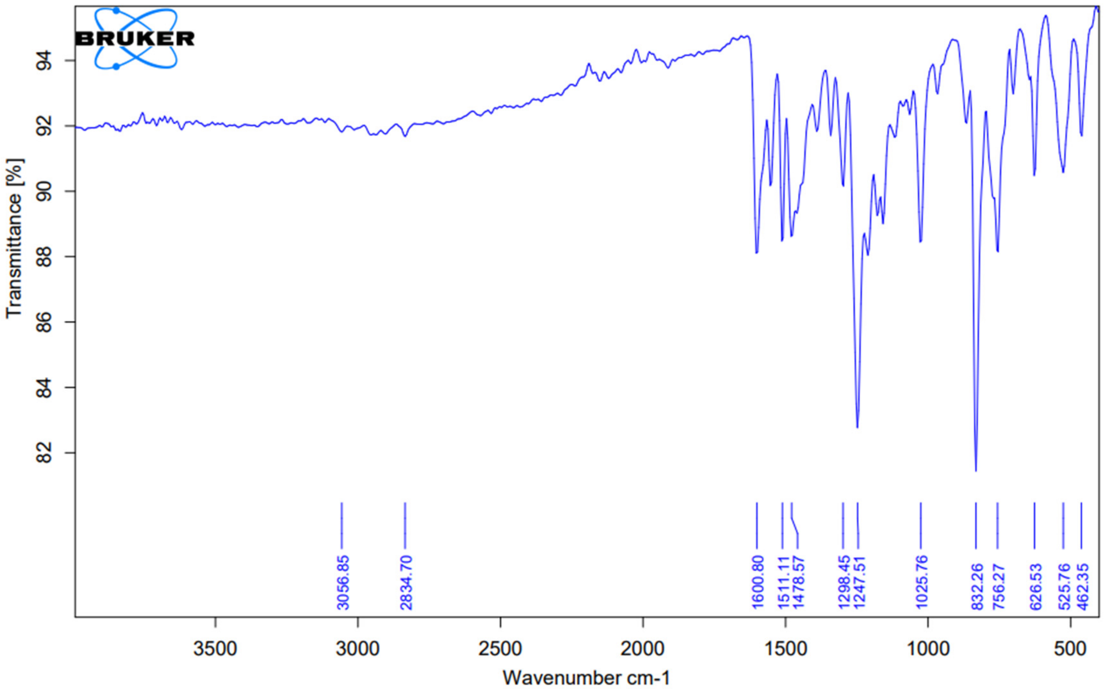

(c)

Figure S1.11. Copies of ^{1}H-NMR (a), ^{13}C-NMR (b) and IR (c) spectra of **2e**, respectively.

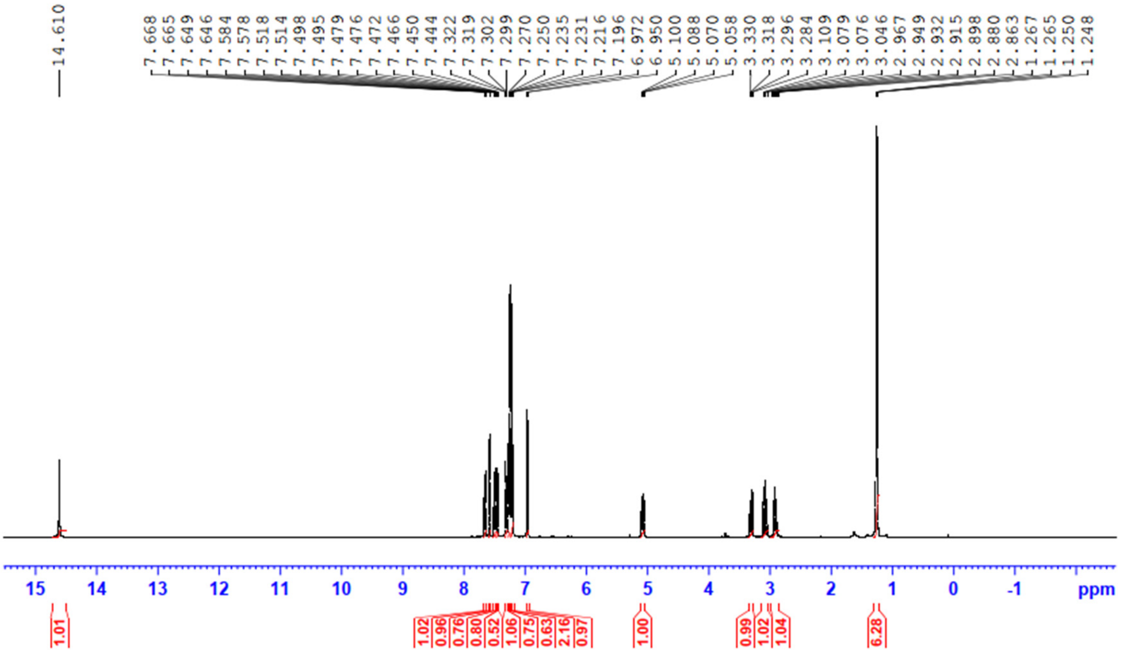

(a)

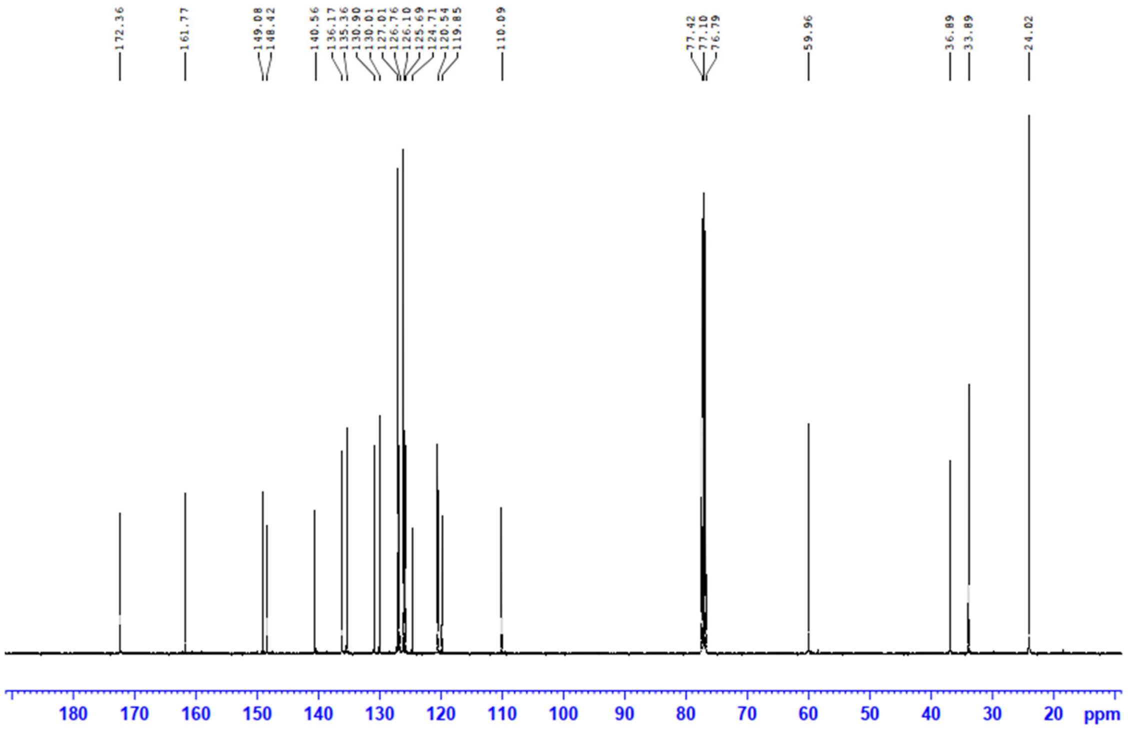

(b)

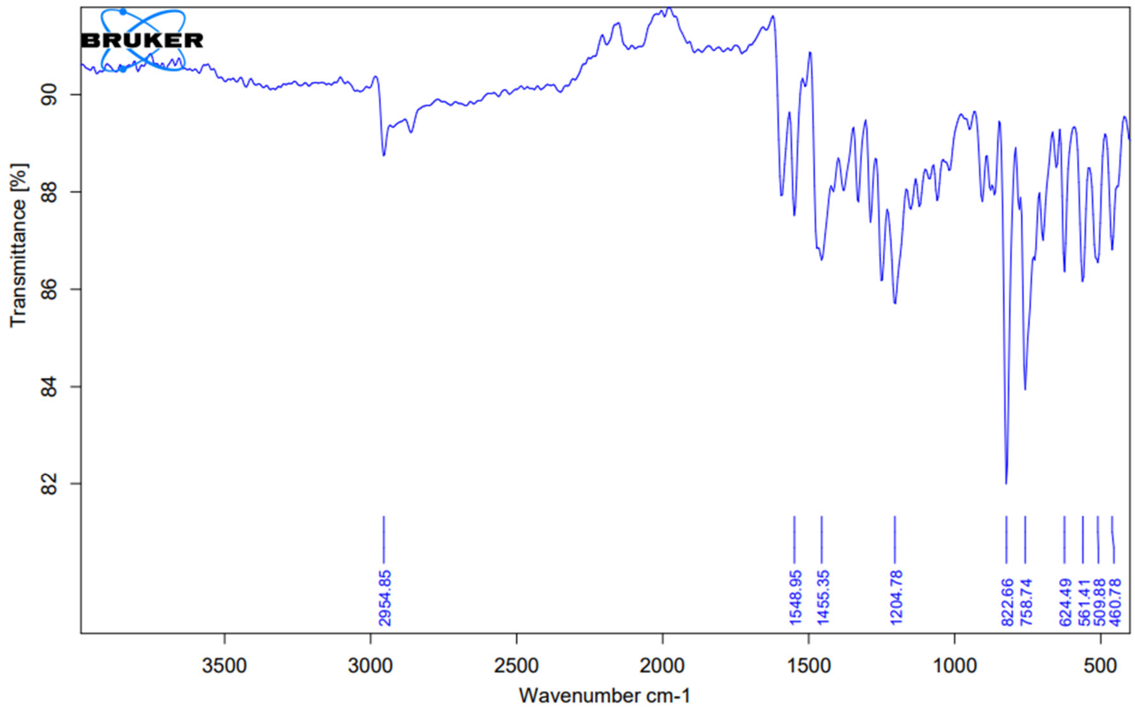

(c)

Figure S1.12. Copies of ¹H-NMR (a), ¹³C-NMR (b) and IR (c) spectra of **2f**, respectively.

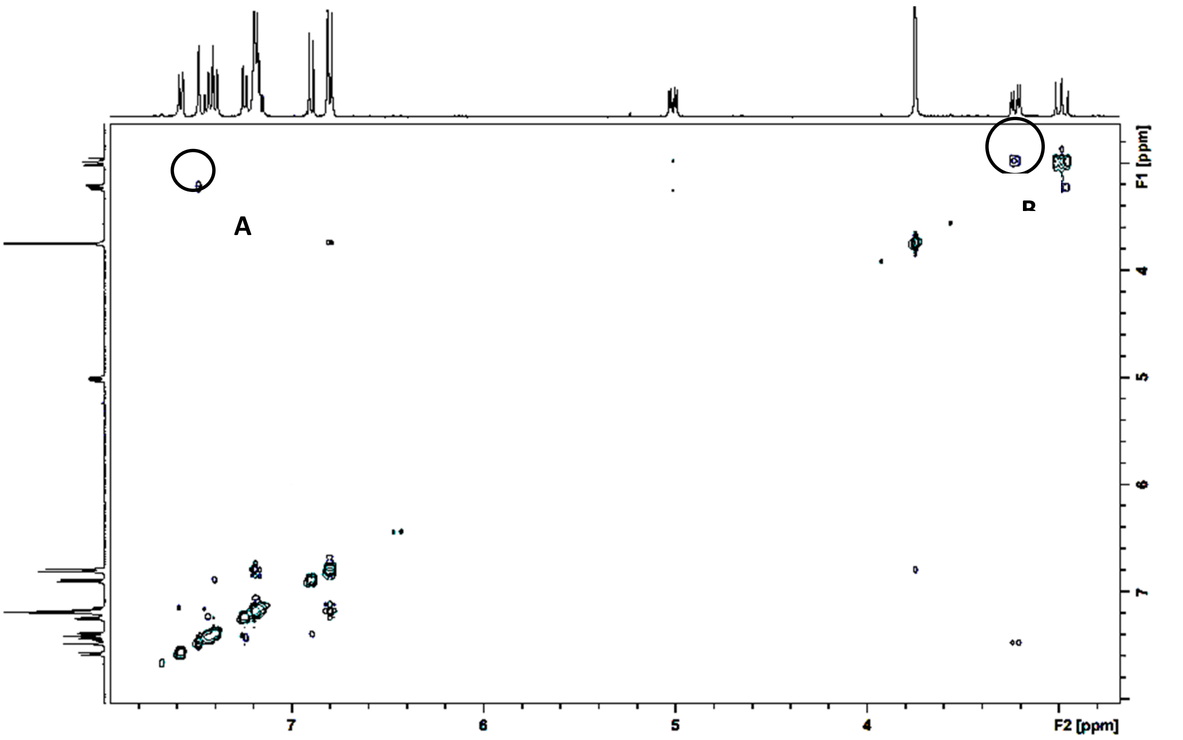

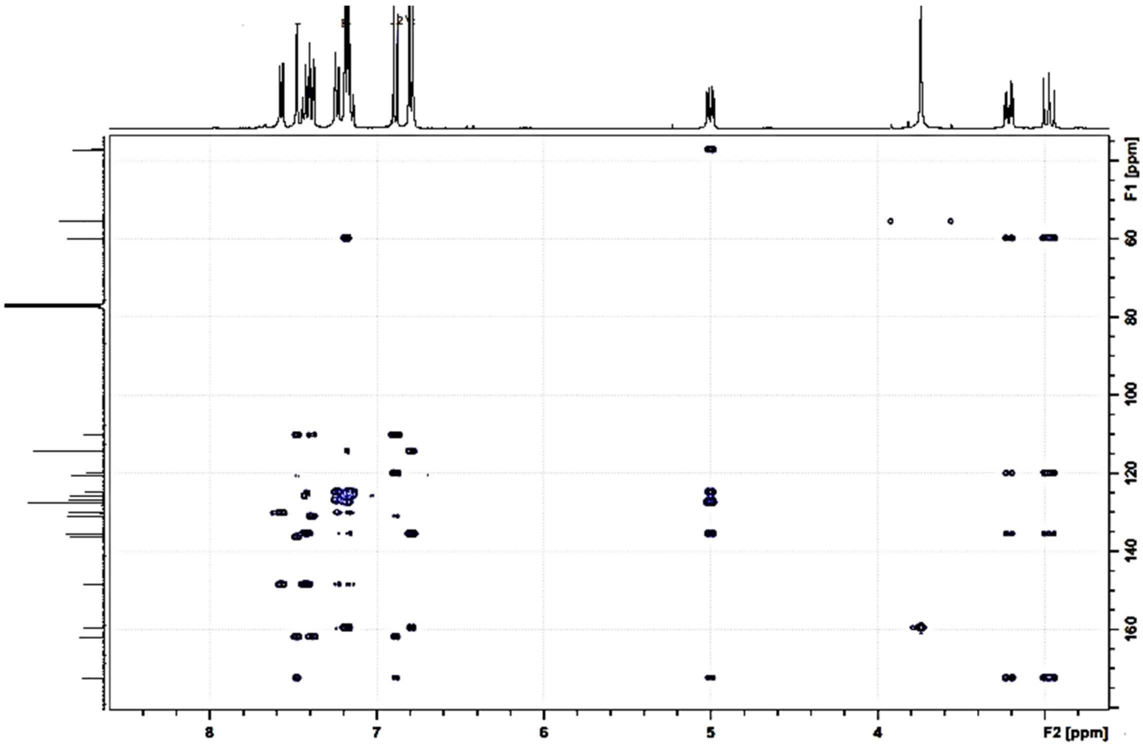

(**b**)

Figure S2. 2D NOESY (**a**) and HMBC (**b**) spectra of **2d** in $CDCl_3$ at 400 MHz

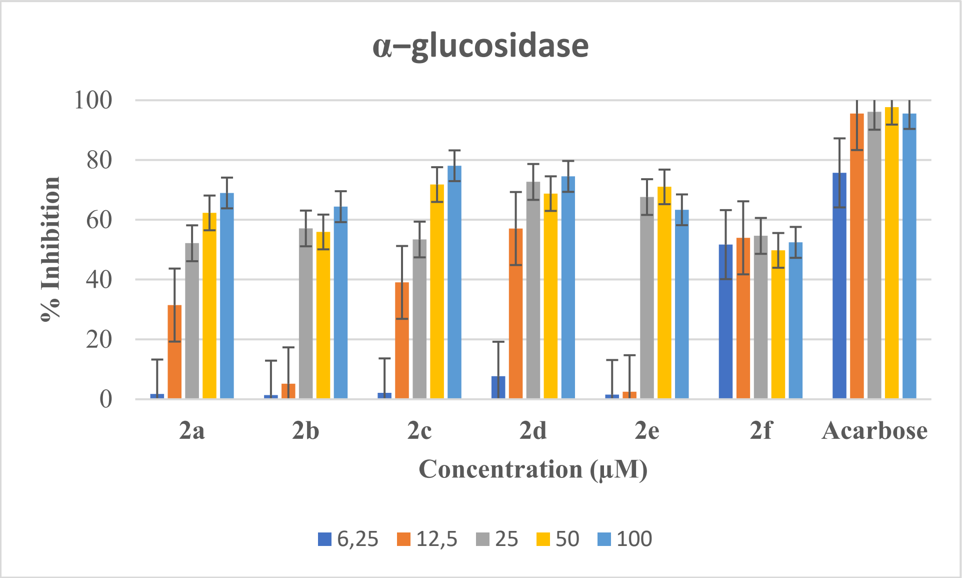

Figure S4. Percentage inhibition of α-glucosidase by compounds **2a–2f** and acarbose.

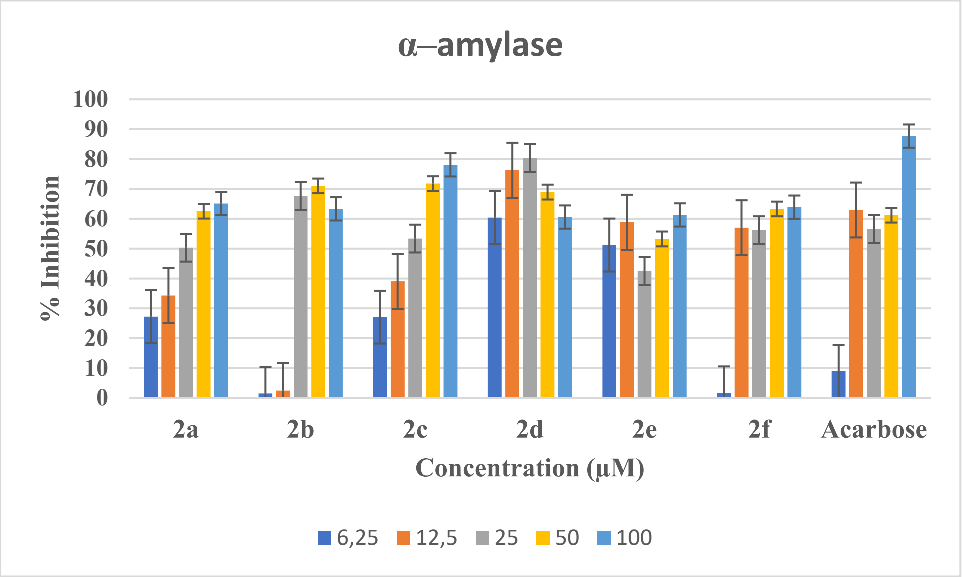

Figure S4. Percentage inhibition of α-amylase by compounds **2a–2f** and acarbose.

Table S1. Crystal data and structure refinement for **2b**.

Empirical formula	$C_{21}H_{15}NOFSBr$
Formula weight	428.31
Crystal system	Monoclinic
Space group	*C*2/c
a/Å	38.785(2)
b/Å	4.6894(3)
c/Å	19.9934(13)
α/°	90
β/°	99.974(2)
γ/°	90
Volume/Å^3	3581.4(4)
Z	8
ρ_{calc}g/cm^3	1.589
μ/mm^{-1}	2.432
F(000)	1728.0
Crystal size/mm^3	0.559 × 0.175 × 0.028
2Θ range for data collection/°	6.4 to 50.992
Index ranges	$-46 \le h \le 46$, $-5 \le k \le 5$, $-24 \le l \le 23$
Reflections collected	17549
Independent reflections	3295 [R_{int} = 0.0471, R_{sigma} = 0.0416]
Data/restraints/parameters	3295/0/239
Goodness-of-fit on F^2	1.019
Final R indexes [I>=2σ (I)]	R_1 = 0.0459, wR_2 = 0.1108
Final R indexes [all data]	R_1 = 0.0670, wR_2 = 0.1261
Largest diff. peak/hole /e Å^{-3}	1.09/-0.85
